# SERS-Based Detection of Food Contaminants: From Laboratory Sensitivity to Practical Implementation—Bottlenecks and Pathways to Standardization

**DOI:** 10.3390/foods15173152

**Published:** 2026-09-05

**Authors:** Donglin Cui, Xin Zhou, Zuqi Zhou, Jun Sun, Yao Tang, Kunshan Yao

**Affiliations:** 1 School of Electrical and Information Engineering, Jiangsu University, Zhenjiang 212013, China; cuidl@stmail.ujs.edu.cn (D.C.);; 2 School of Low-Altitude Technology, Jiangsu University, Zhenjiang 212013, China; 3 School of Electrical and Information Engineering, Changzhou Institute of Technology, Changzhou 213032, China

**Keywords:** SERS, pesticide residues, mycotoxins, heavy metals, food safety, SERS substrates, chemometrics, machine learning, explainable AI (XAI), matrix effects

## Abstract

Ensuring food safety requires the detection of trace-level contaminants such as pesticides, mycotoxins, and heavy metals. Analytical approaches for these analytes should feature high sensitivity, good selectivity, and compatibility with aqueous matrices; surface-enhanced Raman spectroscopy (SERS) satisfies these requirements. Addressing the absence of a unified comparative analytical framework, this critical review surveys recent SERS-enabled sensing strategies for food contaminants. Detection strategies differ substantially across the three contaminant classes: pesticides can be directly detected at ppb levels through substrate engineering and deep learning; mycotoxins rely on affinity-recognition elements to reach pg-mL-level sensitivity; and Raman-inactive heavy metals demand indirect readout via functional probes. Crucially, despite these divergent analytical routes, the field confronts three shared bottlenecks—spectral irreproducibility, severe matrix interference, and the lack of standardized protocols, all of which hinder regulatory adoption. Compared with near-infrared spectroscopy (NIR) and hyperspectral imaging (HSI), SERS delivers outstanding sensitivity for confirmatory trace-level analysis, while its limited throughput may be compensated by multispectral data fusion. Future advances should prioritize portable sensing hardware, explainable Artificial Intelligence (AI), and multiplexed detection to transfer laboratory-scale sensitivity toward practical field-deployable testing tools.

## 1. Introduction

Global pesticide application reached 2.7 million tonnes in 2020, up from 1.7 million in 1990 [1]. Mycotoxins contaminate an estimated 25% of global cereal production annually [2]; aflatoxin B1 (AFB1) is a Group 1 carcinogen [3]. Globally, 14–17% of cropland is contaminated by toxic metals including lead, cadmium, and arsenic, exposing up to 1.4 billion people to elevated health risks [4]. Pesticides, mycotoxins, and heavy metals are three of the most critical chemical contaminants in food. Pesticides secure yields but leave residues in fruits, vegetables, and cereals. Heavy metals (lead, cadmium, mercury, arsenic) are distributed through industrial emissions and agrochemical applications, and are distinguished by their nondegradability and bioaccumulative nature [5].

Despite their chemical diversity, these three contaminant classes share trace occurrence and chronic toxicity. Rapid, sensitive, and reliable detection methods are therefore required. Conventional methods (GC, LC, their MS variants, and ELISA) are sensitive and selective, but they require costly instrumentation, lengthy sample preparation, skilled operators, and are not field-deployable. Raman spectroscopy delivers vibrational fingerprints: different chemical groups (C-C, C=C, C-H, O-H, S-S) produce distinct shifts, giving it high specificity [6]. Water’s Raman signal is weak, so drying or extraction steps, required for infrared spectroscopy, can be avoided. But the scattering cross-section is intrinsically small: one inelastic event per 10^6^–10^8^ photons, too weak for trace detection without enhancement [7,8].

This limitation has pushed the field toward enhancement strategies, with SERS emerging as the dominant solution. Plasmonic nanostructures, typically gold or silver, amplify Raman signals by factors of 10^5^–10^10^ [9]. The enhancement arises from two synergistic mechanisms: electromagnetic enhancement via localized surface plasmon resonance, which generates intense “hot spots” at nanoscale junctions, and chemical enhancement via charge transfer between adsorbed molecules and the metal surface. Five decades on, SERS has found its way into food safety, environmental monitoring, and biomedical diagnostics as a practical analytical tool [10].

Over the past five years, substantial advances have been made in SERS-based detection across all three contaminant classes. Chemometrics and machine learning now provide systematic solutions for spectral interpretation, covering preprocessing, feature extraction, and model construction [11,12]. For pesticide residues, substrate design (metasurfaces, multivariate optimization, hydrogel hybrids, flexible films, fiber-optic probes) has enhanced both sensitivity and reproducibility. Deep learning methods have enabled multi-residue simultaneous detection through spectral deconvolution of mixed pesticide systems [6,13]. For mycotoxins, detection strategies have moved from direct adsorption to affinity-based formats (aptamers, antibodies, molecularly imprinted polymers), pushing detection limits to pg/mL levels [14,15]. Heavy metal detection, by contrast, poses a distinct challenge, as metal ions inherently lack Raman activity; indirect strategies employing functionalized probes have achieved sensitivity at fM level [16].

Several recent reviews have addressed specific aspects of this field, including reproducibility challenges in pesticide SERS analysis, machine learning-assisted spectroscopic detection of mycotoxins, and heavy metal ion detection strategies using SERS [6,17]. Few existing critical reviews have built a unified comparative analytical framework covering all three contaminant groups. They rarely examine how divergent physicochemical properties drive distinct SERS measurement schemes, while jointly dissecting common practical bottlenecks that hinder regulatory translation in real-world practice. This gap is particularly striking because such a framework could explain why these techniques, despite decades of research, have not yet become routine tools in food safety monitoring.

Pesticides, mycotoxins, and heavy metals are selected for this comparison not only because they represent high-priority hazards for food safety. They also form a logical gradient in the complexity of Raman detection. Despite large physicochemical differences among them, these analytes share identical practical barriers that hold back SERS deployment in real-world practice. This common ground renders such side-by-side analytical comparison meaningful for a critical review.

As a critical review, this work addresses that gap by constructing a comparative analytical framework organized around a central question: how can SERS sensitivity achieved in the laboratory be translated into methods suitable for field deployment and accepted by regulatory bodies? We show that the three contaminant classes form a gradient of increasing strategic complexity. Pesticides can be detected directly. Mycotoxins require sensing elements for recognition. Heavy metals must be detected indirectly through functionalized probes. Yet despite these divergent strategies, all three converge on the same translational bottlenecks: poor spectral reproducibility, severe matrix interference, and the absence of standardized protocols. By critically evaluating current solutions and identifying actionable pathways, this review aims to offer not just a reference but also a forward-looking roadmap for moving SERS beyond proof-of-concept studies and toward practical food safety surveillance.

## 2. The SERS Toolbox

### 2.1. Principles of Raman Scattering

Trace-level detection of food contaminants is a problem of molecular identification. Raman spectroscopy addresses this through inelastic light scattering. Upon monochromatic irradiation, most photons scatter elastically with no frequency shift, whereas a tiny fraction (roughly one in 10^6^ to 10^8^ incident photons) scatters inelastically, yielding vibrational fingerprints characteristic of specific chemical bonds (e.g., C-C, C=C, C-H, O-H) [7,8].

For food analysis, this technique offers a crucial practical advantage: because the Raman signal of water is exceptionally weak, aqueous samples can be measured directly without drying or extraction steps [8]. However, the intrinsically small scattering cross-section precludes trace detection without enhancement. Over decades, this limitation has pushed the field toward enhancement strategies, with SERS emerging as the dominant solution [7,18].

### 2.2. Surface-Enhanced Raman Spectroscopy

When molecules are adsorbed onto nanostructured Au, Ag, or Cu surfaces, Raman signals can be amplified by orders of magnitude. That amplification is the phenomenon known as SERS. Fleischmann et al. first observed it in 1974, though the underlying mechanism, localized surface plasmon resonance (LSPR), was not clarified until later. Five decades on, SERS has found its way into food safety, environmental monitoring, and biomedical diagnostics as a practical surface-analytical tool [19].

Two mechanisms work together to produce the SERS effect [19,20]. Electromagnetic (EM) enhancement comes from LSPR, which creates “hot spots”: intensely localized fields that boost the excitation at the adsorbate. EM typically provides 10^4^–10^10^-fold enhancement and dominates the total gain due to its long-range nature (2–5 nm). To tune the resonance, precise control over the size, shape, and spacing of the nanoparticles is required, parameters routinely optimized through FDTD or DDA simulations [21].

**Figure 1:**
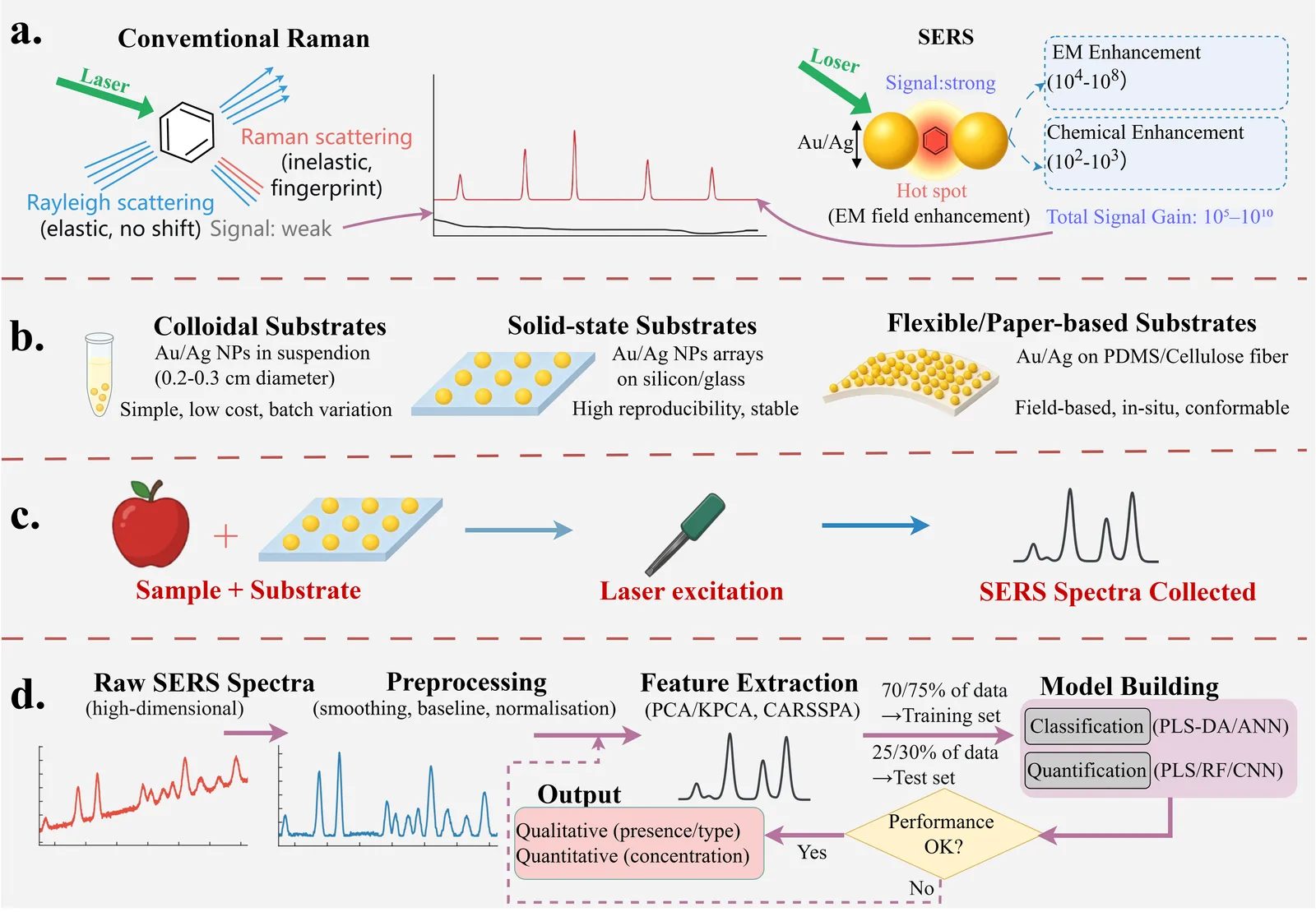
Overview of the SERS analytical workflow for food contaminant detection. The figure addresses four sequential questions: (**a**) why SERS is needed (i.e., enhancement over conventional Raman); (**b**) which substrates are used (colloidal, solid-state, or flexible/paper-based); (**c**) how the SERS signal is acquired (laser excitation of sample-loaded substrates); and (**d**) how the spectral data are processed.

Chemical (CM) enhancement, meanwhile, involves charge transfer between the adsorbed molecule and the metal surface. The resulting charge-transfer complexes change the molecular polarizability over a short range (1–5 Å) and add roughly 10^2^–10^3^-fold enhancement. The overall SERS enhancement factor typically ranges from 10^5^ to 10^10^, with extreme values approaching 10^11^ under optimized conditions. It should be noted that these values are calculated using different definitions and experimental conditions, making direct cross-study comparisons unreliable. While such extreme sensitivity can enable single-molecule detection in ideal laboratory settings, this achievement is not representative of routine food contaminant analysis [22]. The enhancement of SERS over conventional Raman is illustrated in Figure 1a.

### 2.3. SERS Substrates: The Core Determinant of Detection Performance

Among the factors governing SERS performance, the substrate is a critical determinant of enhancement, spectral quality, and reproducibility [23]. This influence, however, does not operate in isolation; analyte adsorption, sample matrix, laser parameters, acquisition conditions, and instrument configuration also contribute substantially. For substrate selection, this framework provides clear guidance: colloids offer simplicity but suffer from uncontrolled aggregation; solid-state substrates provide uniformity and robustness at the cost of more complex fabrication; and flexible substrates provide a practical compromise for field sampling. An optimal substrate would offer high enhancement, uniform hot spots, chemical stability, and consistent fabrication [23].

Colloidal Au, Ag, and Cu nanoparticles remain the most widely used SERS materials, though solid-state and flexible formats are gaining ground. Silver gives the strongest signal, but it tarnishes readily; gold is more stable and biocompatible but less enhancing [23,24]. Particle shape also matters: spheres produce even fields, while sharp-tipped structures (nanostars, nanoflowers, nanoprisms) create intense local spots. Gaps between particles (≤10 nm), the well-known “hot spots”, amplify fields through plasmonic coupling: smaller gaps mean stronger signals [25].

The second category is solid-state substrates, where nanoparticles are attached to silicon, glass, paper, or polymer films. Colloids are cheap and simple to prepare, but aggregation is hard to control; ionic strength, pH, and solvent composition all affect it. This leads to batch-to-batch variability, a major source of SERS’s notorious irreproducibility [24,26]. Many researchers have shifted away from colloids for quantitative analysis. Solid-state formats sacrifice some simplicity in exchange for better stability and reproducibility [25].

A third class, flexible paper- or PDMS-based substrates, has appeared more recently. These materials conform to uneven surfaces and allow direct swabbing or adhesion-based sample collection, which is useful for field work. Paper-based substrates, especially, are inexpensive, biodegradable, and easily functionalized. They have already been applied to pesticides and mycotoxins [27,28]. Beyond these established categories, newer designs include core–shell particles (Au@Ag, SiO_2_@Au), hydrogel-embedded networks, and dielectric-supported structures. These can improve stability and hot-spot density, but their performance in real food samples remains poorly documented [25,29]. The three main classes of SERS substrates are summarized in Figure 1b, and the spectral acquisition procedure is shown in Figure 1c.

### 2.4. Chemometrics and Machine Learning

Raw SERS spectra are high-dimensional: hundreds of data points per measurement, which poses challenges. Overlapping bands from multiple contaminants lead to spectral congestion, where the signal at any given wavenumber is a mixture of contributions from several analytes, matrix components, and noise. Traditional univariate calibration, based on a single peak, is not reliable in this situation because the peak may not be specific to the target. This is where chemometrics and machine learning come in, typically through three steps: preprocessing, feature extraction, and model building [23,30].

Preprocessing choices have consequences. Poor baseline correction, for example, can inject errors that carry through to the final prediction. Optimizing these parameters for specific analyte–matrix systems deserves more attention than it usually receives.

Even after preprocessing, hundreds of variables remain, many of them redundant or noisy. Feature reduction helps, either by extracting latent components (PCA) or by selecting informative variables (CARS, SPA). PCA projects the data onto orthogonal axes that capture maximum variance; for non-linear structures, kernel PCA is more appropriate [31]. Selection methods like CARS and SPA identify the most useful wavenumbers, which reduces model complexity and improves predictive accuracy.

The choice of model depends on the analytical task. Classification models (LDA, PLS-DA, SIMCA, ANN) determine whether a contaminant is present and what type it is, whereas quantitative models (PLS, PCR, random forest) estimate concentrations [23]. For mycotoxins at pg/mL levels, the signal-to-noise ratio is marginal, so quantitative models must be validated against matrix-matched standards; otherwise, sensitivity can be overestimated.

Deep learning has transformed the landscape because it automatically extracts complex non-linear features, which is advantageous for heavily overlapped spectra. For instance, the integration of a hierarchical porous chiral SERS substrate with a fully connected neural network (FCNN) enabled accurate discrimination of mixed pesticide residues [32]. In parallel, data fusion strategies boost predictive performance; PLSR models based on Raman and near-infrared spectral fusion significantly improved aflatoxin B1 detection [33].

To prevent overfitting and ensure generalizability, rigorous validation protocols are required. Datasets should be systematically split into training and test sets (e.g., via the Kennard-Stone algorithm), and external validation on independent datasets is essential. For model tuning, stratified k-fold cross-validation provides a robust balance between accuracy and efficiency [34]. Issues of class imbalance, where contaminated samples are scarce, should be addressed through data augmentation or class weighting to avoid biased predictions [35].

Furthermore, regulatory acceptance is hindered by deep learning’s black-box nature. Since food safety decisions can lead to the destruction of contaminated batches, models must be transparent and explainable to support high-stakes decisions. Explainable AI (XAI) is becoming the gold standard for decision support systems. Tools such as SHAP and LIME enable researchers to identify which spectral features drive quantitative outputs, thereby facilitating regulatory acceptance [36,37]. The entire chemometric workflow is shown in Figure 1d.

### 2.5. Search Strategy

Literature retrieval for this critical review was performed using two complementary academic databases, covering articles published up to August 2026. The Web of Science Core Collection was used as the primary retrieval database, covering core academic literature indexed in the Science Citation Index (SCI) and Engineering Index (EI). The Bohr Academic platform was adopted as a supplementary retrieval source to capture regional foreign language journals, conference papers, and dissertations not included in the Web of Science Core Collection, so as to fill the retrieval blind spots of a single database. Consistent search terms were used across both platforms, combining analytical technique descriptors (“surface-enhanced Raman spectroscopy” or “SERS”) with target contaminant keywords including “pesticides”, “pesticide residues”, “mycotoxins”, and “heavy metals”. These keyword sets were further supplemented by analytical terms “detection”, “sensing”, and “analysis”. Only English-language publications were considered in the primary database search. Full-text access to all retrieved literature was obtained through institutional authorization via the university campus network and VPN service.

Duplicate records from the two databases were removed first, followed by screening of titles and abstracts to exclude papers with weak relevance to food-related contaminant detection. Full-text assessment was then carried out for the remaining candidate papers, with particular attention paid to experimental evidence obtained from actual food matrices. Beyond the initial search results, reference lists of key published reviews were manually inspected to retrieve additional representative studies that might not be captured by the keyword search.

As a critical review, this work does not seek to include every single available publication on the subject. Article selection prioritized studies offering tangible insights into SERS sensing schemes, substrate performance characteristics, measured analytical detection performance, or practical translational obstacles for food contaminant testing. Papers reporting only proof-of-principle results purely in buffer solutions were included only when they delivered essential mechanistic understanding for subsequent investigations in food contexts.

## 3. Analytical Strategies for Three Contaminant Classes

### 3.1. Pesticide Residues: Confronting the Reproducibility Crisis and Multi-Residue Complexity

Pesticide residues are most frequently detected as multi-residue contamination in fruits and vegetables [38]. Beyond direct toxicity, pesticide residues have been increasingly studied in conjunction with antimicrobial resistance and microbial contamination in food safety research [39]. These two concerns have intensified the demand for rapid, sensitive, and reliable pesticide monitoring in food supply chains. Recent advances in SERS-based detection of organophosphorus pesticides have been systematically reviewed, with particular emphasis on substrate engineering and the relationship between nanomaterial design and molecular enhancement mechanisms [40]. Conventional analytical methods, including gas chromatography, liquid chromatography, their tandem mass spectrometric variants, and enzyme-linked immunosorbent assays, offer high sensitivity and selectivity [41]. However, their practical utility is constrained by protracted analysis times, complex sample preparation, and substantial instrumentation costs. SERS addresses the sensitivity barrier through plasmonic amplification. Yet sensitivity is no longer the primary impediment in pesticide detection. Two more intractable challenges have come to dominate the field: spectral irreproducibility across laboratories and spectral congestion arising from co-contaminating pesticide mixtures. The origins of these challenges, and the strategies developed to address them, are examined below.

#### 3.1.1. Origins of the Reproducibility Crisis

To contextualize the irreproducibility of SERS-based pesticide detection, it is instructive to consider conventional Raman spectroscopy. Free from substrate–molecule interactions, conventional Raman yields reproducible spectral patterns that serve as the reference baseline for SERS band assignment. The conventional Raman spectra of malathion, chlorpyrifos, and imidacloprid are characterized by distinct vibrational signatures. Chlorpyrifos, for instance, exhibits SERS peaks at 341, 412, 678, 1092, and 1571 cm^−1^ in tomato matrices, aligning with its conventional Raman spectrum [40]. SERS spectra of the same pesticide, however, vary considerably across studies. This phenomenon constitutes the core of the reproducibility crisis [42].

The reproducibility crisis originates from three substrate-related factors. Distinct metal compositions yield different localized surface plasmon resonance conditions, altering the spectral response for the same analyte. Morphological variations modulate hot-spot distribution, leading to signal fluctuations that are difficult to control. Poorly controlled aggregation introduces batch-to-batch signal variability. These factors are not independent; aggregation changes the effective interparticle spacing, which in turn modifies the plasmonic coupling and thus the observed enhancement factor. The combined effect is that two laboratories using nominally identical protocols can report substantially different SERS spectra for the same pesticide.

A deeper and less-explored question is why SERS substrate design lacks a unifying theoretical framework analogous to the van Deemter equation in chromatography, which relates plate height to flow rate and particle size. Chromatography benefits from a well-established theoretical foundation that guides method development and enables cross-laboratory comparison. SERS substrate development, by contrast, remains largely empirical. Each research group optimizes its own protocols, but there is no shared basis for comparison across studies. Without such a framework, the field accumulates disconnected reports rather than developing a coherent understanding of substrate–analyte interactions. This empirical mode of development has generated a vast diversity of substrate designs, but it has also perpetuated the reproducibility crisis.

Colloidal substrates, despite offering simple preparation procedures, suffer from poorly controlled aggregation. The aggregation state is influenced by ionic strength, pH, and solvent composition, all of which vary across sample matrices and experimental conditions. Solid-state substrates, in contrast, provide superior mechanical stability and operational reproducibility, though they often require more complex fabrication procedures [23]. The trade-off between simplicity and reproducibility is a recurring theme in SERS substrate design.

#### 3.1.2. Substrate Engineering for Uniform Hot Spots

Several emerging substrate designs have been developed to circumvent the limitations of colloidal suspensions, with the shared overarching goal of generating uniform, reproducible hot spots for consistent SERS enhancement. Existing solutions can be broadly categorized by their hotspot-stabilization mechanism: rigid structural patterning, soft matrix confinement, and flexible surface adaptation.

Rigid structural patterning employs solid scaffolds to fix nanoparticle spatial positions. Architectures of this type reduce random nanoparticle aggregation, though fabrication complexity remains a practical trade-off. For example, ZnO nanorod/Ag nanoparticle-functionalized paper substrates delivered an enhancement factor of 9.8 × 10^6^ for thiabendazole and outperformed substrates modified solely with silver nanoparticles [43]. Periodic hexagonal honeycomb metasurfaces likewise yield well-defined hot-spot regions less susceptible to batch-to-batch variation, enabling sub-ppm detection of multiple fungicides within cucumber extracts [44].

Soft-matrix confinement immobilizes nanoparticles inside hydrophilic polymer networks. This strategy suppresses aggregation-derived signal drift without fully blocking analyte access to plasmonic surfaces. Plasmonic hydrogel hybrids represent one representative implementation, achieving combined improvements in sensitivity, reproducibility, and operational stability [45]. Optimized via factorial and Box–Behnken experimental designs, rGO/AgNP thin-film substrates also follow this stabilization principle and reach approximately 21,500-fold enhancement for ametryn measured on real fruit peels [46].

Flexible surface-adaptable substrates are particularly suited for on-site sampling on uneven food surfaces and eliminate tedious pre-extraction steps. Flexible platforms fabricated from paper, PDMS, or cellulose support direct swab-sampling of produce surfaces. For instance, flexible SERS sensors attained a thiabendazole LOD of 10^−8^ mg/mL on cherry peel surfaces [47]. Complementary demonstrations include SiO_2_@Ag core–shell substrates for ten-minute simultaneous dual-pesticide detection in tobacco leaves [48], alongside PEG-modified gold nanoparticles coupled with PCA for selective neonicotinoid discrimination [49].

Across these families of substrate architectures, the core common principle remains spatial and temporal stabilization of plasmonic hot spots to mitigate signal variability originating from uncontrolled nanoparticle aggregation. A key remaining practical barrier lies in lowering fabrication complexity, so that these reproducibility-improved substrates can move closer toward standardized, regulatory-oriented analytical workflows.

#### 3.1.3. Matrix Adaptation and in Situ Sampling Strategies

Sample matrix constitutes another major source of measurement uncertainty that directly impairs SERS reproducibility. Cuticular wax, plant pigments and pectin generate interfering fluorescence backgrounds and compete for adsorption sites on plasmonic surfaces. Processed commodities such as tea and rice bring additional analytical challenges, as endogenous matrix components interfere both with analyte extraction and spectral acquisition [50]. Such matrix effects are far from trivial; they largely explain the frequent large gaps between LOD values obtained in ideal buffer systems versus those achievable in authentic food samples.

Diverse sampling workflows have therefore been tailored to cope with variable food-matrix properties. Extraction-based protocols such as QuEChERS remove matrix interferents while concentrating target analytes. Combined with handheld Raman devices, this workflow enabled multi-pesticide detection in basmati rice below 10 ppb residue limits [51] and shortened chlorpyrifos sample pretreatment for tea analysis to 15 min [52].

In-situ detection avoids sample transfer steps and minimizes analyte loss. Optical-fiber SERS probes immersed directly into tea infusions achieved rapid screening for thiram (1.0 μg/kg) and paraquat (10.0 μg/kg) [53]. Floating hollow silica-microsphere@AuNP substrates adopt a different in-situ strategy, self-assembling at oil-water interfaces to mitigate aggregation artefacts in multi-phase food-related systems [54].

Surface-swabbing approaches take advantage of flexible and paper-based substrates to collect analytes directly from irregular food surfaces. For leaf-vegetable matrices, 50 nm AuNP substrates enabled simultaneous measurement of phosmet, thiabendazole, and acetamiprid with satisfactory recoveries ranging from 87.67 to 113.75% [55]. Chitosan-modified filter-paper substrates delivered chlorpyrifos detection in wheat at 0.000558 mg/L, with performance comparable to more elaborate solid-state SERS platforms [56]. Complementing discrete point measurements, spray-deposited SERS substrates coupled with imaging can map pesticide spatial distribution over produce surfaces and yield quantitative results with R^2^ = 0.9983—a capability difficult to realize using conventional chromatographic techniques [57].

Taken together, these matrix-adapted sampling strategies constitute indispensable practical prerequisites if SERS pesticide detection is to advance beyond laboratory proof-of-concept toward real-world screening and future regulatory method validation.

#### 3.1.4. Chemometrics and AI for Spectral Deconvolution

The chemometric workflow for SERS-based pesticide detection proceeds through three essential stages: preprocessing, feature reduction, and model construction. Preprocessing includes smoothing to reduce high-frequency noise, baseline correction to remove fluorescence and scattering backgrounds, and normalization to account for signal intensity variations across measurements. These steps are not neutral; the choice of preprocessing parameters can systematically affect subsequent analysis, and suboptimal preprocessing may introduce errors that propagate through the entire workflow. For this reason, the selection of preprocessing strategies must be tailored to the specific analyte–matrix combination.

Feature reduction techniques address the high-dimensionality of SERS spectra. Principal component analysis (PCA) linearly projects the data onto orthogonal axes that capture maximum variance. For non-linear structures, kernel PCA (KPCA) provides a more appropriate representation. Variable selection algorithms, including competitive adaptive reweighted sampling (CARS) and successive projection algorithm (SPA), identify the most informative wavenumbers, thereby reducing model complexity and improving predictive accuracy. For pesticide multi-residue detection, feature reduction is essential for isolating analyte-specific signals from overlapping spectral backgrounds.

Model construction converts the selected features into qualitative or quantitative predictions. Classification models, including linear discriminant analysis (LDA), partial least squares discriminant analysis (PLS-DA), and soft independent modeling of class analogy (SIMCA), determine whether a target pesticide is present and, if so, to which type it belongs. Quantitative models, primarily partial least squares regression (PLS) and random forest regression, predict analyte concentrations.

Three-dimensional SERS substrates based on silver nanoparticles compound polyacrylonitrile nanohump arrays have been fabricated for carbendazim detection, with the BOSS-PLS method achieving a correlation coefficient of 0.992 and recoveries of 86% to 116% in apple samples [58]. Silver nanoflower-based SERS sensors coupled with ICPA-PLS have enabled simultaneous detection of chlorpyrifos and carbendazim in rice, with correlation coefficients of 0.9802 and 0.9995, respectively [59]. Signal-optimized flower-like silver nanoparticles with an enhancement factor of 1.39 × 10^6^ have been deployed for pesticide sensing in green tea, achieving detection limits of 5.58 × 10^−4^ μg/mL for methomyl, 1.88 × 10^−4^ μg/mL for acetamiprid, and 4.72 × 10^−3^ μg/mL for 2,4-D [60]. CARS-PLS for chlorpyrifos in tea used only 1.7% of available spectral variables yet achieved SVM R^2^ = 0.981 [52], demonstrating that parsimonious models can outperform full-spectrum approaches when variable selection is properly executed.

Deep learning has markedly enhanced multi-residue detection capabilities. MC-CNN-GRU (multi-channel convolutional neural network with gated recurrent unit) models achieved 99% accuracy for mixed chlorpyrifos and pyrimethanil detection on fruit surfaces [61]. CNN-based analysis of fruit peels achieved high classification and prediction accuracy for multiple pesticides [57]. Portable Raman spectroscopy combined with CNN has also been successfully applied to pesticide identification [61], indicating that deep learning architectures are particularly well-suited to the challenges of portable, real-time SERS analysis. Density functional theory (DFT) calculations have further revealed competitive adsorption of thiabendazole and pymetrozine on silver surfaces, providing a molecular-level explanation for signal competition in mixed pesticide systems [62].

A critical observation from these studies is that the choice of chemometric method can mitigate, but cannot eliminate, the effects of substrate irreproducibility. Models built using data acquired on one type of SERS substrate or within a single laboratory often fail to generalize to new experimental set-ups. This practical limitation highlights the urgent need for standardized reference spectra and cross-laboratory calibration datasets.

To alleviate this bottleneck, inter-laboratory cross-testing is highly desirable alongside strict validation workflows, including stratified k-fold cross-validation and external validation using fully independent sample datasets. Transfer learning represents another promising workaround for the shortage of high-quality real-world food-sample SERS data; CNN-based transfer-learning frameworks have already proven effective for quantitative SERS measurements across variable measurement conditions [63,64].

Beyond SERS food-safety research itself, useful practical insights may be drawn from adjacent agri-food research domains. Recent reviews of vibrational-spectroscopy applications for plant stress phenotyping show that surface-oriented Raman/SERS analysis developed for plant tissues offers transferable lessons for evaluating complex food matrices [65]. In addition, multimodal data integration, such as the combination of NIR-fluorescence and SERS, can strengthen analytical performance for complicated food matrices and constitutes a promising direction for future food-safety screening workflows [66]. Table 1 summarizes representative SERS-based methods for pesticide residue detection in food matrices, highlighting the substrate configurations and chemometric models that have enabled ppb-level sensitivity across various commodities.

#### 3.1.5. Persistent Challenges and Outlook

Beyond the three cross-cutting bottlenecks already discussed in the introduction, pesticide detection presents additional difficulties in multi-residue scenarios. Spectral congestion from co-contaminating pesticides is a recurring problem, especially when chemically similar compounds coexist in a single sample. Overlapping signals hinder both qualitative identification and accurate quantification, and univariate calibration alone is rarely sufficient for such complex mixtures.

Recent progress in substrate engineering and deep learning-based deconvolution has provided partial relief. Rationally designed substrates with well-controlled hot-spot distributions can reduce measurement variability, and interpretable deep learning models offer a way to identify which spectral features are most relevant for classification or quantification. These approaches are encouraging, but their adoption in routine practice depends on standardized reporting and cross-laboratory validation. The shift toward multi-residue monitoring also calls for portable devices and multiplexed platforms that can deliver reliable performance outside the laboratory.

### 3.2. Mycotoxins: Engineering Biorecognition and Signal Amplification for Ultratrace Analysis

Mycotoxins, secondary metabolites produced by Aspergillus, Penicillium, and Fusarium species, are among the most significant natural contaminants in food and animal feed. Aflatoxin B1 (AFB1), a Group 1 carcinogen, is approximately ten times more toxic than potassium cyanide and predominantly contaminates peanuts, corn, and rice [3]. Other mycotoxins of major concern include ochratoxin A (OTA), which exhibits nephrotoxicity; deoxynivalenol (DON), which induces emesis and immunosuppression; zearalenone (ZEN), which possesses estrogenic activity; and fumonisins, which are associated with esophageal cancer. The severe toxicity of these compounds has prompted regulatory agencies to establish strict maximum residue limits (MRLs), necessitating analytical methods capable of reliable detection at ultratrace concentrations. Approximately 25% of global cereal production is contaminated by mycotoxins annually [2].

The health risks posed by mycotoxins are compounded by their occurrence patterns. Multiple mycotoxins frequently co-occur in the same commodity, and their combined effects may be additive or synergistic. Moreover, mycotoxin contamination is often unevenly distributed within a batch, complicating representative sampling. These characteristics distinguish mycotoxin detection from pesticide detection: the challenge is not only sensitivity but also the ability to detect multiple structurally related toxins simultaneously in a heterogeneous matrix.

Conventional detection methods, including high-performance liquid chromatography (HPLC), liquid chromatography–tandem mass spectrometry (LC-MS/MS), and ELISA, offer high sensitivity but are constrained by expensive instrumentation and protracted analysis times [68]. Recent research advances in SERS-based sensing platforms for multiplex mycotoxin detection have been reviewed, with emphasis on enhancement mechanisms, substrate types, and detection technologies [69]. SERS addresses the sensitivity barrier through plasmonic amplification [68], while biorecognition elements confer the specificity needed to distinguish target mycotoxins from matrix interferents [27,28]. However, the ultratrace concentrations of mycotoxins in complex cereal and oil matrices, combined with the structural similarity among toxin analogues, often limit direct SERS detection. In many applications, recognition or amplification strategies are advantageous to achieve the required sensitivity. The evolution of these strategies, from direct adsorption to affinity-based sensing and signal amplification, is examined below.

#### 3.2.1. Recognition Elements

Direct adsorption of mycotoxins onto SERS substrates has been demonstrated in several studies. Ag-pSi substrates achieved 0.0085 ppb AFB1, with aptasensors outperforming immunosensors in reproducibility (7 versus 1 regeneration cycle) [70]. Signal-enhanced SERS sensors using silver nanoparticles coupled with CAR-PLS and GA-PLS algorithms have been developed for ochratoxin A and aflatoxin B1 detection. The limits of detection were 2.63 pg/mL for OTA and 4.15 pg/mL for AFB1 in spiked cocoa beans [71]. PHEMA/AgNP hybrids achieved 10^−9^ M OTA detection [42]. However, the weak adsorption affinity of mycotoxins, combined with fierce competition from matrix components for surface binding sites, renders direct detection insufficient for real-world applications [68]. This limitation has driven the evolution toward recognition-based strategies, in which biorecognition elements selectively capture target mycotoxins and bring them into proximity with the SERS substrate.

Aptamer-based sensors constitute the most extensively developed approach for mycotoxin detection. Aptamers offer several advantages over antibodies: they are synthetically accessible, exhibit batch-to-batch consistency, and can be readily modified with functional groups for immobilization or signal reporting [27,72,73]. AFB1 detection has been achieved through multiple architectural configurations, including photoaffinity-oriented antibody immobilization, SERS/EIS dual-channel formats, and core-satellite self-assembly [74,75,76]. A magnetic-plasmonic and membrane-like nanotag assembly has been developed for aflatoxin B1 detection. The aptasensor exhibited a detection limit of 2.31 pg/mL and a linear range of 10^−2^ to 10^3^ ng/mL, with relative standard deviations of 1.19% to 2.52% [77]. The membrane-like SERS nanotag, formed by assembling a dense layer of AuNPs onto graphene oxide nanosheets, provides signal amplification through interparticle nanogaps of less than 3 nm. These diverse architectures reflect a field that has prioritized sensitivity above all else; the question of which architecture is most suitable for routine deployment remains open.

For applications requiring even higher sensitivity, AgMOF single-atom catalysts (0.002 μg/L) and SERS-fluorescence dual-mode formats (0.0092/0.0050 ng/mL) represent the current performance frontier [78,79]. Gold nanoflower-based sensors (0.03 ng/mL) offer a less sensitive but simpler alternative [80]. The diversity of architectures is a sign of a field in rapid development, but it also indicates that no single approach has yet achieved the combination of sensitivity, reproducibility, and simplicity required for routine application.

Immunosensors and molecularly imprinted polymers (MIPs) offer complementary advantages to aptamer-based sensors. SERS-lateral flow immunosensors achieved 8.9 pg/mL AFB1 with simultaneous detection of imidacloprid and pyraclostrobin [81]. MIPs offer superior stability and reproducibility compared with biological recognition elements, with affinity magnetic beads enhancing both enrichment and sensitivity [82]. MIP-SERS integration remains promising for complex matrices, though the affinity of MIPs for their targets is generally lower than that of antibodies [83,84].

A critical comparison across these recognition elements reveals a clear trade-off that extends beyond analytical performance to practical utility. Aptamers excel in batch consistency and modification flexibility but may lack affinity for certain targets; their synthesis cost is moderate and preparation time is short (days to weeks) [72,73]. Antibodies provide superior affinity and specificity but suffer from poor stability, limited regeneration (often single-use), higher cost, and batch-to-batch variability [68,81]. MIPs offer unmatched chemical and thermal stability, low cost, and reusability, but their affinity improvements are still needed; their preparation is time-consuming (weeks to months), and current protocols are not yet standardized [82,83]. For practical deployment, the choice of recognition element must consider not only LOD but also cost per assay, shelf life, matrix compatibility, and regulatory acceptance. These dimensions are rarely systematically compared in the literature.

#### 3.2.2. Signal Amplification Strategies

Signal amplification is the second indispensable pillar of mycotoxin SERS detection. The ultratrace concentrations of mycotoxins, often in the pg/mL range, are below the direct detection limit of even the best SERS substrates without amplification. Two broad classes of amplification strategies have been developed: nanomaterial-based cascade amplification and nucleic acid-based amplification.

Nanomaterial cascade amplification offers moderate gain with straightforward implementation. Au@Ag core–shell nanoparticles and AgMOF single-atom catalysts provide enhanced SERS signals through a combination of plasmonic enhancement and catalytic amplification [78]. The core–shell architecture reduces the distance between the analyte and the plasmonic surface, while the catalytic activity of the shell material can generate additional signal-enhancing species. These strategies are relatively simple to implement and are compatible with standard SERS substrate fabrication protocols.

Nucleic acid amplification provides substantially higher gain at the cost of greater operational complexity. CRISPR/Cas12a-based detection achieved 0.79 pg/mL AFB1 [3], while CRISPR-SERS strategies reached 3.55 pg/mL AFB1 [84]. The CRISPR system offers sequence-specific recognition coupled with enzymatic signal amplification, enabling detection limits that are competitive with conventional chromatographic methods. The trade-off, however, is substantial: CRISPR-based assays require multiple enzymatic steps, precise temperature control, and trained personnel. These factors limit their field deployability.

Dual-mode readouts have emerged as a strategy to enhance reliability through self-validation. SERS-fluorescence dual-mode sensors [79] and SERS/EIS dual-channel sensors [75] provide two independent detection channels on a single platform. A flexible paper-based SERS sensor using flower-like silver nanoparticles has been developed for chloramphenicol detection. The MSC-CARS-PLS model achieved a limit of detection of 10^−5^ μg/mL and recoveries ranging from 90% to 102% in real samples [85]. Discrepancies between the two channels flag potential interferences, reducing false positives in complex matrices. The added complexity of dual-mode detection is justified for high-stakes regulatory applications where false positives are unacceptable. The selection of amplification strategy therefore depends on the application context: for rapid screening, simpler nanomaterial amplification may be sufficient; for confirmatory analysis requiring ultratrace sensitivity, nucleic acid amplification or dual-mode readouts may be necessary.

#### 3.2.3. Matrix-Specific Applications

The performance of mycotoxin detection strategies varies substantially across food matrices, reflecting differences in matrix composition and analyte distribution. In cereals, which account for the majority of mycotoxin contamination incidents, BOSS-PLS achieved R^2^p = 0.9927 for AFB1 in wheat, while CRISPR/Cas12a achieved 0.79 pg/mL AFB1 [3]. The relatively simple matrix composition of cereals, predominantly starch and protein, facilitates extraction and reduces matrix interference, making them the most extensively studied commodity type.

In oils and nuts, the hydrophobic nature of the matrix complicates both extraction and SERS detection. QuEChERS-AuNPs detected 0.00092 ng/g AFB1 in palm oil [86], while CARS-PLS achieved a prediction set determination coefficient of 0.99 for AFB1 in peanut oil [87]. A SERS-based lateral flow immunosensor using core–shell Au@Ag nanoparticles has been developed for zearalenone detection in corn. The detection range was 10 to 1000 μg/kg with a limit of detection of 3.6 μg/kg, far below the recommended tolerable level of 60 μg/kg [88]. CNN feature fusion achieved R^2^p = 0.9836 in peanuts [30], demonstrating that deep learning can compensate for matrix-induced spectral variability. The oil matrix generates strong fluorescence backgrounds and competes for adsorption sites on the SERS substrate, requiring more extensive sample preparation compared with cereal matrices.

In dairy products, AFM1 detection, a metabolite of AFB1 that appears in milk from contaminated livestock, has been reviewed across multiple methods [89]. The protein-rich matrix of milk presents unique challenges, including protein precipitation and nonspecific adsorption. Metal nanoparticle-based sensors have been explored for AFM1 detection, but the sensitivity and specificity of these methods generally lag behind those developed for cereals and oils. The heterogeneity of mycotoxin distribution across different food types underscores the need for matrix-matched calibration standards and validation protocols.

#### 3.2.4. Discrimination of Structurally Similar Analogues

Discrimination of structurally similar toxins presents a distinctive challenge that is largely absent from pesticide detection. AFB1, AFB2, AFG1, and AFG2 share nearly identical coumarin skeletons and produce heavily overlapping SERS spectra [27]. This spectral similarity arises because the Raman scattering cross-sections of these molecules are dominated by the common coumarin framework, while the substituents that differentiate the analogues contribute only weakly to the Raman spectrum.

Pesticides, by contrast, typically involve distinct functional groups (organophosphates, carbamates, triazoles) that produce well-separated Raman bands. Heavy metals circumvent the spectral overlap problem entirely through indirect detection: the SERS signal arises from the probe molecule, not from the metal ion itself, so the spectra of different metals are distinguished by the probe’s response rather than by the metal’s intrinsic vibrations. The mycotoxin case is unique: the toxins are chemically similar, Raman-active, and require both sensitivity and selectivity.

Strategies for discrimination of mycotoxin analogues include recognition element cross-reactivity control and multi-labeling approaches. Cross-reactivity control involves selecting aptamers or antibodies that differentiate closely related analogues based on subtle structural differences. Multi-labeling approaches use different Raman reporters for different targets, enabling multiplexed detection through spectral deconvolution of the reporter signals. The coffee-ring effect has been exploited for the rapid enrichment detection of patulin and alternariol in apples. Partial least squares models based on synergy interval and genetic algorithm variable selection yielded correlation coefficients of 0.9905 for patulin and 0.9829 for alternariol, with limits of detection of 1 μg/L for both toxins [90]. Neither approach is fully satisfactory. Cross-reactivity control is limited by the inherent similarity of the target molecules; multi-labeling increases assay complexity and cost.

This challenge is fundamental, not merely technical. Without a recognition element that distinguishes the analogues or a substrate that selectively adsorbs one analogue over another, the SERS spectrum of a mixture of aflatoxins will be dominated by the most strongly scattering species, regardless of its concentration. This limitation is not unique to SERS; it is shared by other spectroscopic techniques and reflects the intrinsic difficulty of distinguishing structurally similar molecules. The mycotoxin community has therefore focused on developing recognition elements that maximize discrimination rather than on improving spectral deconvolution methods. Table 2 summarizes representative SERS-based methods for mycotoxin detection. Aptamer-based sensors, immunosensors, and CRISPR-amplified strategies together span a detection range from pg/mL to sub-pg/mL across multiple food matrices.

#### 3.2.5. Persistent Challenges and Outlook

Mycotoxin detection shares the cross-cutting bottlenecks outlined in the introduction, but two problems are particularly acute for this class. One is the difficulty of discriminating structurally similar analogues. AFB1, AFB2, AFG1, and AFG2 produce heavily overlapping SERS spectra because their Raman fingerprints are dominated by the common coumarin framework. This is a fundamental challenge. Without recognition elements that can distinguish between these analogues, the SERS spectrum of a mixed sample will be dominated by the most strongly scattering species regardless of its concentration.

The second issue is the limited stability of biorecognition elements. Aptamers and antibodies remain the most effective capture probes, yet their shelf life under ambient conditions is short. They require refrigeration and careful handling, which limits their use outside the laboratory. MIPs offer better stability but are still early in development and generally have lower affinity than biological recognition elements [70,83]. The trade-off is clear: higher affinity often means lower stability, and the reverse is also true.

Future progress will depend on extending the operational lifetime of recognition elements, designing chips that can detect multiple toxins without cross-reactivity, and agreeing on sample preparation protocols that work across different food matrices. The selectivity problem is not unique to SERS, but it is particularly pressing here because the health risks posed by mycotoxins demand reliable identification at very low concentrations.

### 3.3. Heavy Metals: Indirect Probing and the Detect-And-Degrade Paradigm

Heavy metals, including cadmium, lead, mercury, arsenic, and chromium, exhibit pronounced biotoxicity and environmental persistence. Chronic exposure causes renal dysfunction, neurotoxicity, hematopoietic damage, and carcinogenic effects [96]. Unlike pesticides and mycotoxins, heavy metal ions are spectroscopically silent; they lack vibrational fingerprints in the Raman spectral range. This fundamental difference mandates entirely indirect SERS sensing frameworks, in which the SERS signal arises not from the metal ion itself but from a probe molecule whose spectral signature changes upon metal-ion coordination. It should be noted that the term “heavy metals” is scientifically broad. For elements such as arsenic and chromium, toxicity and analytical behavior depend strongly on their chemical species and oxidation states (e.g., As(III)/As(V) and Cr (VI)). As recent reviews emphasize, speciation analysis is critical for environmental monitoring and risk assessment, since the total concentration of a metal (loid) is rarely a good indicator of risk [97]. SERS-based strategies capable of distinguishing chromium species through distinct spectral signatures have been demonstrated, highlighting the potential of this technique for on-site speciation [98]. Therefore, this review places particular emphasis on valence speciation when discussing these elements.

Heavy metals enter the food chain via soil and irrigation water, accumulating in crops, aquatic products, and agricultural commodities. Regulatory frameworks including GB 2762 (China) [99], Codex Alimentarius (international) [100], and EFSA (European) [101] classify lead, cadmium, mercury, and arsenic as priority food contaminants, subject to stringent maximum residue limits across cereals, vegetables, tea, aquatic products, and milk. The widespread occurrence and regulatory stringency have made heavy metal detection a priority for food safety monitoring, but the indirect nature of SERS detection imposes constraints that are absent from the pesticide and mycotoxin cases.

In the field of heavy metal detection, atomic spectrometric techniques, including atomic absorption spectrometry (AAS), atomic fluorescence spectrometry (AFS), inductively coupled plasma optical emission spectrometry (ICP-OES), and inductively coupled plasma mass spectrometry (ICP-MS), remain the most widely applied laboratory standard methods, offering high accuracy and good reproducibility for confirmatory quantification [102,103,104,105]. These techniques require bulky instrumentation, complex sample preparation (typically acid digestion), and skilled operators, and they are incapable of on-site rapid screening or direct speciation analysis. SERS offers complementary advantages: fingerprint recognition through probe molecules, rapid response, portability, and the potential for field-deployable screening.

Given the absence of intrinsic vibrational fingerprints, the methodological landscape for heavy metal SERS detection diverges fundamentally from those of pesticides and mycotoxins. The following sections are organized by strategic complexity, progressing from simple ligand-based recognition to amplified sensing and integrated functionalities. Each approach occupies a distinct position on the trade-off continuum between operational simplicity and analytical sensitivity.

#### 3.3.1. Ligand-Functionalized Substrates

The conceptually simplest indirect strategy immobilizes ligand molecules onto SERS substrates to generate spectral shifts upon metal-ion coordination. Upon metal binding, variations in ligand polarizability, conformation, or molecular orientation produce measurable SERS signal changes. No exogenous reporter is required; the ligand itself acts as the signal transducer. This simplicity makes ligand-functionalized substrates attractive for routine screening applications.

The design logic is well illustrated by the 2-mercaptoisonicotinic acid (2MNA)-AuNP system, which supported simultaneous Hg^2+^ and Pb^2+^ discrimination using characteristic peak intensity ratios: I_1160_/I_1230_ for Hg^2+^ (LOD 3.4 × 10^−8^ M) and I_861_/I_815_ or I_815_/I_1392_ for Pb^2+^ (LOD 1.0 × 10^−7^ M) [106]. Single vibrational peaks could not differentiate the two analytes, whereas combined ratio features delivered adequate discrimination. The reliance on multiple peak ratios rather than a single peak is a recurring theme in heavy metal SERS detection.

A more advanced design utilized GSH and 4-MBA dual-modified Au@Ag core–shell nanorods for Pb^2+^ analysis. The bimetallic architecture combines Au cores for good colloidal dispersity and Ag shells for strong plasmonic enhancement, eliminating the need for DNA or aptamer recognition units. Free carboxylate groups on GSH selectively chelate Pb^2+^ and trigger nanoparticle aggregation, forming abundant hot spots [107]. This design exemplifies how substrate architecture can be tailored to achieve both sensitivity and selectivity without biorecognition elements.

Other ligand-functionalized platforms have been developed for a range of heavy metals and food matrices, including Cd^2+^ in tea powder [108], Hg^2+^ in tea infusions and milk [109], and simultaneous screening of multiple metals in agricultural water [110]. An aptamer-regulated gold nanoparticle reduction method has been developed for lead ion detection in food. The first-order derivative preprocessing combined with competitive adaptive reweighted sampling achieved a correlation coefficient of 0.9972 and a detection limit of 0.1 μg/L [111]. Detailed performance metrics for these methods are provided in Table 3. Ligand-functionalized substrates possess clear practical merits: minimal sample pretreatment and compatibility with portable Raman devices. The trade-off, however, is that achievable sensitivity generally falls within the nM-μM regime, while selectivity is entirely governed by ligand binding affinity.

**Table 3:** Representative SERS-based methods for heavy metal detection in food matrices.

Target Analyte	Food Matrix	SERS Substrate	Recognition Strategy	Matrix Type	Reported LOD	Original Unit	Ref.
Pb^2+^	Tea powder, glutinous rice flour	Au@Ag core–shell nanorods	GSH + 4-MBA modified	Spiked food matrix	0.021	μg/L	[107]
Hg^2+^	Fish meat samples	Spider-web Au nanowire array	4-MBA IS + 4-ATP probe	Spiked food matrix	1.35 × 10^−13^	M	[112]
Hg^2+^	Green tea infusion	Paper chip + 4-MPY-AuNPs	Ag staining amplification	Spiked food matrix	0.48	pM	[109]
Hg^2+^	Drinking water, tuna	Au/Ag bimetallic nanostructures	Cy5-DNA reporter	Spiked food matrix	208.71	fM (water)	[113]
Hg^2+^	Milk, yogurt, milk beverages	Multifunctional AuNPs	4-MBA IS + thymine recognition	Spiked food matrix	1.2	μg/L	[114]
Cd^2+^	Black tea powder	Sodium alginate-green AgNPs	TMT ligand	Spiked food matrix	2.36 × 10^−5^	μg/L	[115]
Total arsenic	Keemun black tea	Cubic Cu_2_O/Ag composite	Label-free As-O-Ag coordination	Spiked food matrix	5.61	ppb	[116]
Cr(VI)	Rice, environmental water	Au@Ag NPs (2 nm Ag shell)	RhB reporter	Spiked food matrix	0.074	nM	[117]
Pb^2+^	Black tea powder	Aptamer-regulated AuNPs	Aptamer-mediated AuNP-generation turn-on sensing	Spiked food matrix	0.1	μg/L	[102]
Hg^2+^, Cd^2+^, Co^2+^, Pb^2+^	Agricultural/irrigation water	Quaternized xylan paper + Au/Ag NPs	4-MBA + R6G probes	Spiked food matrix	Hg^2+^: 10^−15^–10^−3^; Cd^2+^: 10^−9^–10^−3^; Co^2+^: 10^−7^–10^−3^; Pb^2+^: 0.1–100	M/ppm	[108]

GSH, glutathione; 4-MBA, 4-mercaptobenzoic acid; 4-ATP, 4-aminothiophenol; 4-MPY, 4-mercaptopyridine; TMT, trithiocyanuric acid; RhB, rhodamine B; R6G, rhodamine 6G; IS, internal standard. LOD values are reported in the units provided in the original references. Label-free As-O-Ag coordination enables direct speciation of As^3+^ and As^5+^ without chromatographic separation. Multiple target analytes in a single row indicate simultaneous detection.

#### 3.3.2. Photocatalytic Substrates for Integrated Detection and Degradation

A second family of indirect sensors exploits photocatalytic materials capable of both SERS detection and pollutant degradation. Ag_3_PO_4_ microcubes and Ag/Ag_3_PO_4_ fiber probes have achieved Hg^2+^ and Pb^2+^ LODs down to 10^−15^–10^−12^ M with recyclability potential [111,118]. The dual functionality is attractive from a sustainability perspective: the same substrate that detects the contaminant can also eliminate it, reducing waste and enabling substrate reuse.

The detection-degradation dual functionality, however, introduces a fundamental tension. Conditions that optimize stable probe–analyte binding for sensitive SERS readout are not those that favor photocatalytic radical generation for breakdown. Stable binding typically requires mild conditions that preserve the probe–analyte complex; photocatalytic degradation requires energetic conditions (e.g., UV irradiation, reactive oxygen species) that can destroy the complex. Most studies demonstrate both functions sequentially, first detection under mild conditions, then degradation under harsher conditions, without systematic evaluation of whether dual functionality compromises either performance. The sequential demonstration is not equivalent to integrated functionality; the detection step may perturb the substrate in ways that reduce subsequent degradation efficiency, and vice versa.

For food safety applications, where quantification is the primary requirement, photocatalytic substrates are therefore treated in this review as a specialized rather than a mainstream strategy. The added material complexity must be justified by specific application demands, such as the need for substrate reuse in continuous monitoring scenarios. For routine food screening workflows that center on stable, repeatable quantification, the trade-off between dual functionality and analytical performance is not yet justified. The photocatalytic approach represents a promising research direction [111,118], but its practical utility in food safety monitoring remains to be demonstrated through systematic comparative studies.

#### 3.3.3. Nucleic Acid-Mediated Amplification and Digital SERS

Nucleic acids enable programmable molecular recognition coupled with versatile signal amplification schemes. Unlike small-molecule ligands, DNA and RNA sequences can be rationally designed for high metal ion selectivity, while enzymatic or cyclic reactions deliver substantial signal gain. Such architectures push heavy metal SERS detection toward the fM regime, at the expense of more elaborate experimental workflows.

A dual recycling amplification SERS aptasensor attained an outstanding Hg^2+^ LOD of 0.11 fM [119]. A bimodal sensor based on upconversion nanoparticles decorated with gold nanoparticles has been fabricated for dibutyl phthalate detection in food. The fluorescence and SERS methods achieved limits of detection of 0.0087 ng/mL and 0.0108 ng/mL, respectively [120]. Similarly, DNAzyme-initiated rolling circle amplification realized Pb^2+^ detection reaching 3.1 × 10^−17^ M [121]. This sensitivity improvement, approximately six orders of magnitude beyond ligand-based sensors, comes at a cost: multi-step enzymatic incubation, precise thermal control, and trained operators restrict field deployability. The gap between what is achievable in a well-equipped laboratory and what is feasible in routine screening is nowhere wider than in this category. Yet it is precisely this category that offers the most compelling evidence that SERS can match the sensitivity of conventional instrumental methods.

Digital SERS offers an alternative route toward reliable trace analysis. Conventional analogue SERS quantification suffers intrinsically from fluctuations in laser power, heterogeneous hot-spot distribution, and inconsistent sample positioning. These are key components of the reproducibility crisis outlined in pesticide SERS detection. Digital SERS circumvents these drawbacks by translating continuous spectral intensities into binary positive/negative readouts for confined microscale detection volumes. Analyte concentration is statistically calculated via Poisson distribution, decoupling quantification from absolute signal fluctuations. Capillary-confined Ag nanostars implemented this strategy for trace Hg^2+^ measurement, achieving an LOD of 10 ppt (approximately 50 pM) [122]. Separate work constructed 3D hot-spot substrates inside capillaries, delivering a 0.2 pM LOD well below established regulatory limits [123].

Amplification strategies deliver ultra-low LODs but cannot be deployed on-site; digital SERS represents an intermediate solution with superior sensitivity compared with simple ligand sensors and lower operational burden than full biochemical amplification. Existing literature has summarized methods for sample preparation and signal amplification in antibiotic detection using SERS, covering solid-phase extraction, solvent microextraction, microfluidics, lateral flow immunoassays, magnetic enrichment, and molecularly imprinted polymers [124].

#### 3.3.4. Valence Speciation and Advanced Substrate Architectures

Among heavy metal SERS methodologies, valence speciation constitutes a distinctive capability that is difficult to accomplish with conventional atomic spectrometry without chromatographic separation. SERS-enabled redox speciation delivers unique analytical benefits. For arsenic accumulated in rice and cereals, As(III) exhibits toxicity 50–100 times greater than As(V), making speciation indispensable for accurate dietary risk evaluation. The ability to distinguish oxidation states directly, without separation, is one of the strongest arguments for SERS-based heavy metal analysis. Arsenic speciation can be realized label-free via interfacial arsenic–silver interactions [125,126]. Free surface Ag^+^ facilitates As-O-Ag complex formation on AgNPs; differing coordination geometries and bond strengths between As(V) and As(III) produce an approximately 60 cm^−1^ Raman shift difference, with respective LODs of 4.0 × 10^−11^ mol·L^−1^ and 8.0 × 10^−11^ mol·L^−1^. Building on this mechanism, Bi_2_SeO_5_-Ag hybrid substrates reached an enhancement factor of 3.6 × 10^6^ and resolved well-separated peaks near approximately 455 cm^−1^ (As^3+^) and 804 cm^−1^ (As^5+^) [126]. Tested on rice samples, LODs reached 10^−7.37^ M for As(III) and 10^−8.17^ M for As(V). The substrate tolerated interference from over ten foreign ions and maintained stability for 14 days under ambient storage. The label-free approach is particularly attractive because it eliminates the variability introduced by probe molecules.

For Hg^2+^ testing in fish tissue, ratiometric SERS chips constructed from spider-web Au nanowire arrays utilized 4-MBA as internal standard and 4-ATP as responsive probe [112]. Quantitative analysis relied on the intensity ratio I_391_/I_1079_ to correct substrate variation, yielding an LOD of 1.35 × 10^−13^ M across 10^−11^–10^−13^ M (R^2^ = 0.9988). Interconnected porous networks generate 2.3-fold stronger signals than discrete Au nanoparticle films, and ratiometric calibration suppresses signal drift (RSD = 0.97%). In comparison, optimized DNA sequences combined with dual coffee-ring enrichment achieved variable Hg^2+^ performance in tuna: 208.71 fM in pure buffer, rising to 9.87 nM in canned tuna and 79.06 nM in raw tuna [113].

For total arsenic determination in tea, label-free Cu_2_O/Ag composite substrates depend on As-O-Ag coordination without extrinsic reporters [116]. Cu_2_O cores supply charge-transfer enhancement, while deposited Ag nanoparticles contribute electromagnetic plasmonic amplification. The method achieved a LOD of 5.61 ppb over a 1–20 μg/g linear range, with recoveries of 96.63–103.60% and results statistically indistinguishable from ICP-MS (*p* > 0.05). The absence of extrinsic reporters reduces assay complexity and eliminates the variability associated with probe synthesis and conjugation. Table 3 summarizes representative heavy metal detection approaches across diverse food matrices, differentiating methods validated in buffer solutions from those verified in authentic food extracts, and listing LOD, linear range, R^2^, enhancement factor, RSD, and recovery.

#### 3.3.5. Quantitative Comparison Across Strategy Classes

The four technical categories, ligand-functionalized substrates, photocatalytic substrates, nucleic acid amplification, and digital SERS, occupy separate positions on the balance curve between analytical sensitivity and practical operability.

Ligand-functionalized substrates generally deliver nM-μM LODs via straightforward protocols, rendering them suitable for routine screening. Their sensitivity may fail to meet strict regulatory limits for certain metals, but their simplicity and compatibility with portable Raman devices make them attractive for field applications. The trade-off is acceptable when the required LOD is within the nM regime.

Nucleic acid amplification sensors push detection capability toward fM-aM levels, an improvement of 10^3^–10^6^ times over ligand sensors. This sensitivity gain comes at the cost of complex enzymatic workflows and skilled operators. The gap between what is achievable in a well-equipped laboratory and what is feasible in routine screening is widest in this category. These methods are best suited for confirmatory analysis rather than rapid screening.

Photocatalytic substrates support integrated detection and potential reuse, but the trade-offs between sensing and degradation remain insufficiently explored. The dual functionality is attractive from a sustainability perspective, but the inherent tension between the conditions required for each function limits their practical utility for routine food safety monitoring.

Digital SERS provides an intermediate route: improved sensitivity compared with ligand platforms, simpler operation than full biochemical amplification, and enhanced reproducibility via threshold binary counting. The digital approach addresses one of the core reproducibility issues, signal fluctuations, by converting analogue intensity measurements into binary readouts. The practical viability of digital SERS for food safety monitoring will depend on the development of simple, disposable digital SERS devices.

Collectively, the three contaminant families face distinct SERS bottlenecks: pesticide measurement suffers from substrate irreproducibility and spectral overlap; mycotoxin analysis is hindered by ultratrace concentrations and structurally similar analogues; heavy metal detection relies on indirect probe schemes but enables unique elemental speciation that is difficult to achieve with other techniques. Atomic spectroscopic techniques (AAS, ICP-OES, ICP-MS) remain the gold standards for laboratory-based confirmatory analysis, a baseline against which SERS-based strategies should be benchmarked. SERS occupies a complementary niche: rapid on-site screening and redox speciation without chromatographic separation. The quantitative comparison presented in this section is not intended to rank the approaches but to clarify the trade-offs that inform method selection for specific applications.

#### 3.3.6. Persistent Challenges and Outlook

Heavy metal detection by SERS is inherently indirect, which adds a layer of difficulty beyond the common bottlenecks noted in the introduction. The performance of functionalized probes depends critically on ligand selectivity and binding affinity. In real food samples, however, multiple competing metal ions are present, and they can cross-react with the probe or suppress the target signal. Proteins in milk and fish cause nonspecific adsorption on probe surfaces, while humic acids and inorganic salts in agricultural samples interfere with the probe–analyte interaction. Mitigation strategies such as protein precipitation, masking agents, and controlled ionic strength are available, but their effectiveness varies with the matrix, and they cannot be applied universally [69].

Another persistent issue is the stability of probes under realistic storage and operating conditions. Many functionalized substrates lose sensitivity over time, and regenerable substrates that maintain consistent performance across multiple uses are still limited.

Future progress will depend on probes with higher selectivity, substrates that can be reused without significant signal loss, and workflows that balance sample cleanup with the speed advantage of SERS. Machine learning models trained on matrix-matched calibration data may help mitigate interference, but the priority for heavy metals is not lower detection limits, as amplification methods have already reached fM levels. The real need is for probes that remain stable in complex matrices and sensors that can be deployed reliably outside the laboratory. Nanozyme-based strategies have also been explored for food safety applications [127], and other nanostructure-based optical and electrochemical platforms have been reported for food safety monitoring [128].

### 3.4. Cross-Contaminant Comparison: Common Principles and Strategic Divergences

The preceding three sections have examined SERS-based strategies for pesticides, mycotoxins, and heavy metals in turn. The parallel examination reveals a systematic pattern: the three contaminant classes are arranged along a gradient of decreasing intrinsic Raman activity, from pesticides (direct) to mycotoxins (recognition-enhanced) to heavy metals (fully indirect), and each step along this gradient imposes a corresponding increase in strategic complexity. This gradient reflects the fundamental relationship between analyte properties and the analytical strategies required for their detection. To ensure meaningful comparisons, matched analyte–matrix systems are used wherever possible to avoid confounding effects arising from differences in sample matrices.

Two general principles emerge. One is that intrinsic Raman activity is a necessary but not sufficient condition for direct detection; concentration and matrix effects ultimately determine whether recognition-based strategies are required. Another is that the three classes constitute a gradient of increasing strategic complexity and sensitivity gain: pesticides (direct, ppb) to mycotoxins (recognition and amplification, pg/mL) to heavy metals (fully indirect, fM). Each step adds layers of complexity but also enables detection at increasingly lower concentrations.

The most fundamental divergence lies in the information transfer pathway. Pesticide molecules directly “write” vibrational information into the SERS spectrum through their intrinsic Raman scattering. Mycotoxins carry similar information, but their ultratrace concentrations require recognition elements to “pull” them from the background and position them at the hot spots. Heavy metal ions are completely silent and must be “translated” through probe molecules that report the metal’s presence via spectral changes. The probe introduces an extra layer of interpretation absent in the other two cases.

**Figure 2:**
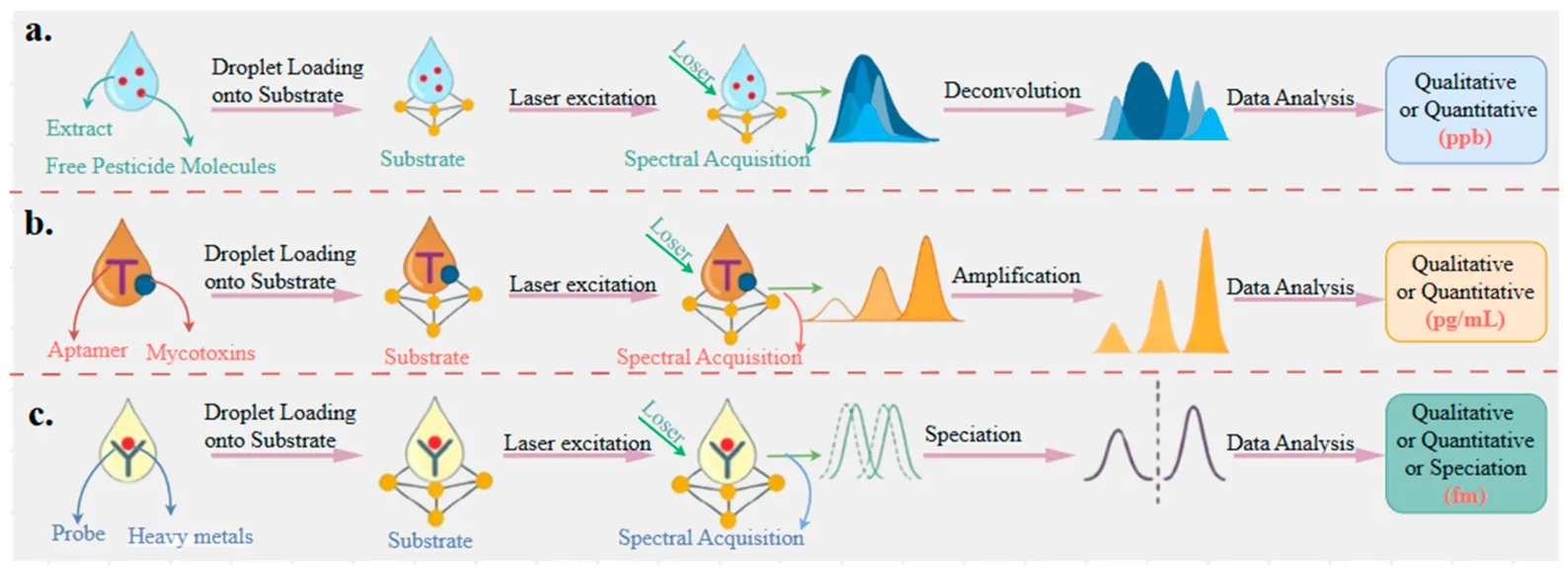
SERS-based strategies for three contaminant classes. (**a**) Pesticides: direct detection of intrinsically Raman-active molecules with spectral deconvolution (ppb). (**b**) Mycotoxins: recognition-element-mediated capture with signal amplification (pg/mL). (**c**) Heavy metals: indirect probing via functionalized reporters for Raman-inactive ions, with valence speciation capability (fM). Strategic complexity increases progressively from (**a**−**c**).

This hierarchy guides strategy selection for new contaminants: first ask whether the target is Raman-active; if not, a fully indirect approach is required. If it is Raman-active, assess whether its concentration and matrix occupancy are sufficient for direct detection; if not, recognition elements may be needed. Figure 2 illustrates these strategic differences schematically, showing the progression from direct detection to recognition-mediated detection to fully indirect detection.

A critical note on reported detection limits is warranted. Many studies report fM or aM LODs achieved in buffer solutions, yet these values may not translate to complex food matrices where competitive adsorption and matrix effects are operative. The difference can be several orders of magnitude. Standardized reporting practices that clearly distinguish LODs obtained in ideal conditions from those demonstrated in real matrices are needed; without such distinctions, cross-study comparisons remain problematic and regulatory acceptance is impeded. Table 4 directly assesses the practical applicability of these laboratory LODs by comparing them against regulatory maximum permissible concentrations (MPCs). While safety margins in real matrices remain exceptionally large for most analytes (e.g., >10^4^-fold for AFB1 and Cd^2+^), they are notably compressed compared to ideal buffer conditions, as strikingly demonstrated by the mycotoxin DON, where the margin shrinks to approximately 117-fold in corn and wheat. Conversely, the QuEChERS extraction in Logan et al. [51] illustrates that optimized sample preparation can actively reverse matrix effects, restoring the safety margin of carbendazim in rice from an insufficient 0.2-fold in buffer to above 1-fold. Therefore, the assessment of SERS sensitivity for routine food control must be based on matrix-matched safety margins rather than absolute LODs in buffer.

Despite the divergences outlined above, three cross-cutting bottlenecks unite the three classes: spectral irreproducibility, matrix interference, and the absence of regulatory-approved protocols. These bottlenecks originate in the inherent complexity of SERS measurement itself: the combination of substrate variability, sample heterogeneity, and environmental sensitivity that makes SERS both powerful and difficult to standardize. Of these, spectral reproducibility and standardization are the most fundamental; without progress on these fronts, the other two bottlenecks become significantly harder to address.

The tools described in this chapter, substrate engineering, recognition elements, amplification strategies, and chemometric deconvolution, are deployed differently across the three contaminant classes, reflecting their distinct physicochemical properties. The shared challenges of reproducibility, matrix interference, and standardization connect the three cases and point toward common solutions. SERS-assisted multimodal biosensing has been integrated with microfluidic, fluorescence, colorimetric, electrochemical, and molecular imprinting technologies to improve analysis accuracy and specificity. These multimodal approaches address the limitations of single-mode detection by providing complementary information and reducing false positives in complex food matrices [132].

## 4. Comparative Analysis with NIR and HSI

SERS does not operate in isolation. NIR spectroscopy and HSI are also widely used for food contaminant detection, each offering distinct trade-offs in sensitivity, throughput, and operational complexity. This chapter positions SERS alongside these techniques. Their physical principles, performance characteristics, and the prospects for strategic integration are examined.

NIR spectroscopy is characterized by low cost, straightforward operation, and minimal sample preparation, making it well-suited to high-throughput screening applications [6,133,134]. HSI integrates spectral and spatial information acquisition, enabling simultaneous characterization of chemical composition and spatial distribution within a single measurement [135,136]. These techniques are governed by distinct physical principles that define their respective performance boundaries. Raman/SERS achieves the highest sensitivity but requires complex substrate fabrication [50]; NIR is operationally the simplest but sensitivity-limited [133]; HSI provides the most information-rich output but generates large data volumes and requires expensive instrumentation [137]. Recognition of these complementary relationships is a prerequisite to informed technique selection for specific detection scenarios.

### 4.1. Physical Principles and Information Content

Raman spectroscopy probes molecular vibrational modes through inelastic light scattering, producing sharp, chemically specific spectral bands that serve as molecular fingerprints [7]. SERS amplifies these signals through plasmonic enhancement, pushing detection limits to ppb-fM levels while retaining fingerprint specificity [5,113,116,138]. The information content is both structurally diagnostic and quantitatively precise, provided that reproducible substrates and optimal acquisition conditions are achieved [139].

NIR spectroscopy, by contrast, measures overtones and combination bands of fundamental molecular vibrations, primarily C-H, O-H, and N-H stretching modes [140,141,142]. These bands are broad, heavily overlapped, and less chemically specific than Raman fingerprints. The information is predominantly statistical rather than structural, requiring multivariate calibration to extract quantitative or qualitative conclusions [87]. NIR signals arise from all components in the sample matrix simultaneously, making spectral interpretation more susceptible to matrix variation. The advantage of rapid data acquisition without reagents comes at the cost of reduced specificity and the requirement for extensive, matrix-dependent calibration models [30].

HSI combines spectroscopy with imaging, capturing a full spectrum at each pixel in the image plane. The technique generates a three-dimensional data cube: two spatial dimensions (x, y) and one spectral dimension (λ) [128,132,135]. This structure enables the simultaneous capture of spectral information about chemical composition and spatial information about where that composition is located. The data processing challenges introduced by this structure include high dimensionality, spatial autocorrelation, and the need for dimensionality reduction before classification or quantification. In the context of food contaminant detection, fluorescence HSI is most commonly employed, though Raman-HSI and NIR-HSI configurations also exist. The information content is both chemical and spatial, a capability that neither Raman nor NIR can provide in a single measurement [136]. This richness, however, comes with high data volumes and complex processing requirements.

### 4.2. Performance Trade-Offs

No single technique excels across all dimensions of analytical performance. Each offers a distinct combination of capabilities and limitations that must be weighed against the specific demands of the application.

Sensitivity is the sharpest differentiator. Raman/SERS consistently achieves the lowest detection limits across all three contaminant classes, ppb-ppt for pesticides, pg/mL for mycotoxins, and fM for heavy metals [5,16,137]. This extraordinary sensitivity comes at a cost: substrate preparation, longer measurement times, and higher operational demands [50]. NIR operates at ppm levels, suitable for screening but insufficient for confirmatory trace analysis [140,141]. HSI falls between the two, with sensitivity generally at ppm levels but with the added advantage of spatial contextualization [135,136]. The trade-off is therefore not simply about which technique is more sensitive, but about whether the application demands trace-level certainty or can tolerate screening-level information. It is worth emphasizing that such performance figures are strongly conditioned by the target analyte, food matrix, and method-validation maturity. For this reason, the most appropriate analytical technique should be judged case-by-case.

**Figure 3:**
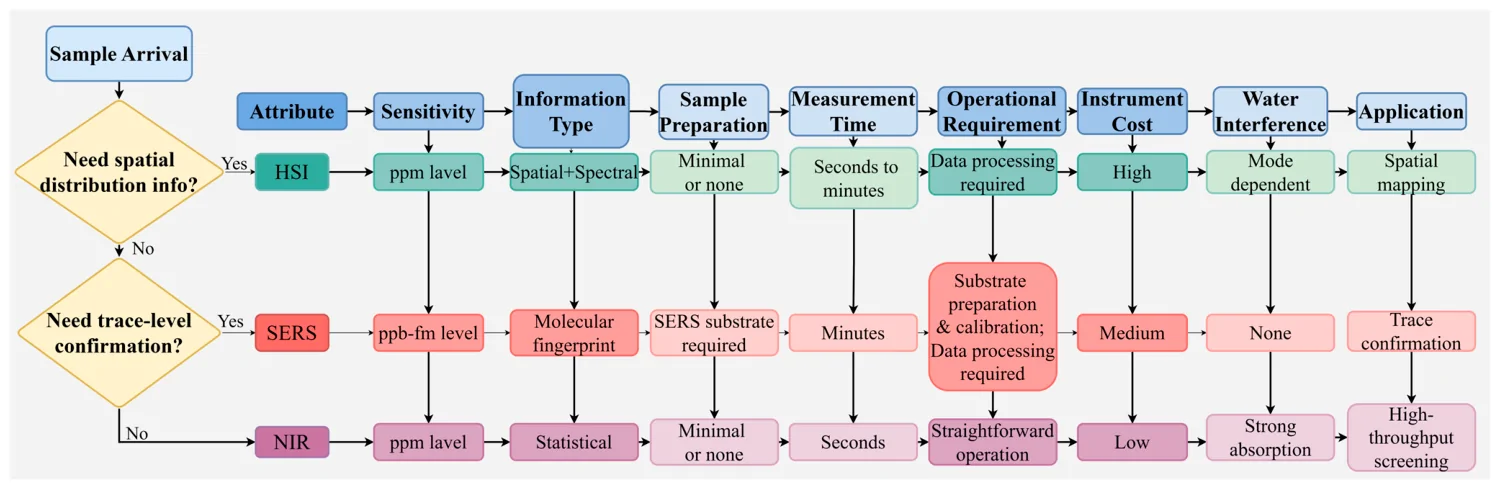
Decision framework for selecting SERS, NIR, or HSI based on detection priority (trace-level confirmation vs. spatial mapping vs. high-throughput screening) and analytical requirements (sensitivity, information type, operational complexity, and cost). HSI: hyperspectral imaging; SERS: surface-enhanced Raman spectroscopy; NIR: near-infrared spectroscopy; ppb: parts per billion; fM: femtomolar; ppm: parts per million.

Throughput, cost, and operational complexity introduce additional differentiation among the three techniques. NIR enables rapid scanning of large sample batches; handheld devices acquire spectra in seconds, making it the method of choice for high-throughput screening [142]. Practical field-oriented vibrational-spectroscopy workflows combining miniaturized spectrometers and chemometric models have been widely explored for food contaminants, especially mycotoxin monitoring [143,144]. HSI requires longer acquisition times but offers richer data per measurement [128,133]. SERS imposes the highest per-sample time cost due to substrate deposition, drying, and measurement steps, though advances in flexible substrates and handheld devices are narrowing this gap [92,134]. Instrumentation costs follow a similar pattern: NIR devices are the most affordable and widely available; HSI systems are the most expensive, requiring sophisticated optics and detectors; SERS instrumentation falls in between, with substrate fabrication as the dominant cost driver, though paper-based and flexible substrates offer an increasingly viable low-cost alternative [134,135]. Operational demands differ correspondingly: NIR requires minimal training, HSI demands significant expertise in data processing, and SERS requires the highest level of skill across substrate preparation, spectral acquisition, and chemometric analysis [132]. Integrated substrate (spectrometer) algorithm systems are gradually reducing this burden [92]. Figure 3 summarizes these trade-offs. The radar plot is not meant to rank the techniques but to illustrate the complementary nature of their performance profiles, a visual reminder that technique selection depends on analytical priority, not technical superiority.

### 4.3. Application Scenarios and Fusion Strategies

The trade-offs described above translate directly into application-specific preferences. Three scenarios illustrate the decision logic.

Scenario 1: High-throughput screening and field-based preliminary inspection. For large-batch screening of grain shipments, fresh produce, or processed commodities where regulatory limits are at ppm levels, NIR spectroscopy offers an optimal balance of speed, cost, and adequate sensitivity [145]. This applies to both laboratory-based screening and on-site field detection, including border inspection, farm-level quality assessment, and retail surveillance. Portable NIR devices are currently the most practical solution due to their low cost, simplicity, and robustness. SERS is gaining ground in this domain with the development of handheld Raman spectrometers and flexible substrates, but field stability and reproducibility of substrates in ambient conditions remain challenges [85,146].

Scenario 2: Confirmatory trace analysis at ppb or lower. For high-risk commodities such as peanuts (AFB1), rice (heavy metals), and fresh produce (multi-residue pesticides), SERS is particularly advantageous. Its unmatched sensitivity and molecular specificity provide definitive identification and quantification that cannot be achieved with NIR alone [116]. SERS is particularly advantageous when analyte identity must be confirmed through characteristic spectral fingerprints rather than inferred through statistical correlation. The higher cost and lower throughput are justified by the analytical certainty required for regulatory enforcement and risk assessment. It is important to recognize, however, that SERS functions best as a complementary tool alongside established chromatographic and mass-spectrometric methods (e.g., LC-MS/MS), which remain the gold standard for regulatory confirmation.

Scenario 3: Spatial distribution mapping. For applications requiring visualization of contaminant heterogeneity, including mapping aflatoxin distribution within a single grain kernel, visualizing pesticide penetration through fruit peel, or localizing fungal infection sites, HSI is uniquely capable. It provides information that neither NIR nor SERS can deliver, albeit at the cost of higher instrument expense and data complexity.

In practice, these scenarios are not mutually exclusive; a single monitoring programme may require all three capabilities at different stages. Many food safety monitoring programmes employ a tiered approach: rapid NIR screening followed by SERS confirmatory analysis for positive samples. This hybrid workflow leverages the throughput of NIR and the sensitivity of SERS, balancing cost and analytical certainty. The same principle extends to multi-spectral fusion, where data from multiple techniques are integrated at the feature or decision level. Feature-level fusion of NIR and Raman spectra into CNN models outperformed either technique alone for AFB1 detection in peanuts [30]. NIR-SERS integration similarly enhanced pesticide residue detection accuracy [147]. HSI-SERS fusion offers a conceptually promising pathway: HSI could identify contaminant hotspots in large samples, enabling targeted SERS measurements at specific spatial positions, substantially reducing substrate consumption while maintaining sensitivity [148]. The primary challenge lies in the spatial co-registration of HSI and SERS data, where different sampling geometries and spot sizes complicate direct comparison.

### 4.4. Positioning SERS in the Spectroscopic Landscape

Across the three techniques, SERS occupies a unique and, for certain tasks, highly advantageous position.

Its first distinguishing feature is molecular specificity. Raman spectra provide sharp, fingerprint-like vibrational information that allows unambiguous identification of analytes, a capability NIR lacks due to its broad, overlapping bands and reliance on statistical correlation [114,149]. This makes SERS particularly useful when identity confirmation is paramount, not just concentration estimation.

Its second advantage is sensitivity. SERS achieves detection limits 2–6 orders of magnitude lower than NIR and generally lower than HSI, making it highly capable of reliable confirmatory analysis at ppb and sub-ppb levels [12,20,23,44,50,150].

Its third, often overlooked, advantage is aqueous compatibility. The inherently weak Raman signal of water, unlike NIR’s strong water absorption, enables direct analysis of aqueous samples without drying or extraction, a significant practical advantage for food matrices such as beverages, sauces, and dairy products [13,126].

These three features, specificity, sensitivity, and aqueous compatibility, collectively define SERS’s niche: it is particularly suitable for confirmatory trace analysis when analyte identity must be established with certainty in complex, often aqueous, food matrices [20,26]. NIR serves the complementary role of high-throughput screening, where speed and cost outweigh the need for definitive identification [114,128,136,151]. HSI serves the specialized role of spatial mapping, where distribution information adds value beyond chemical identification [115,116]. The fusion of SERS with NIR and HSI represents not merely an incremental improvement but a strategic move toward holistic, reliable, and practical food safety monitoring systems [113,138,152,153].

It is crucial to recognize that SERS does not operate in isolation. Rather than replacing established chromatographic and mass-spectrometric methods, which remain the gold standard for ultimate regulatory confirmation, SERS functions as a powerful complementary tool for rapid, on-site, and cost-effective preliminary screening. For a food safety laboratory with limited budget but high sample throughput, NIR is the first-line screening tool [154,155]; for regulatory enforcement requiring legal defensibility of results, SERS confirmatory analysis plays a critical supporting role [12,16,134]. The choice is not about which technique is better, but about which trade-off aligns with the analytical priority.

## 5. Conclusions and Future Perspectives

### 5.1. Persistent Cross-System Limitations

Extensive progress has been achieved in developing SERS sensing methods for pesticides, mycotoxins and heavy metals in food matrices, yet these laboratory-scale achievements cannot be readily transferred to routine industrial food safety monitoring [38,50,134]. Three universal technical hurdles restrict the large-scale deployment of SERS platforms for all three contaminant categories discussed in this review.

Spectral irreproducibility constitutes the most fundamental barrier to comparable, trustworthy SERS measurement data. Even when testing identical analytes, published spectra across independent studies display obvious discrepancies in peak positions, signal intensities and characteristic band assignments [17,26]. Such inconsistency arises from multiple intertwined factors. Variations in nanoparticle composition, geometric morphology, and aggregation behavior alter localized surface plasmon resonance responses, thereby causing inconsistent signal amplification effects [13,21,38,128]. Differences in molecular adsorption conformations and metal-molecule bonding interactions, together with variable laser parameters (wavelength, power and integration duration), further amplify measurement deviations [156]. Without standardized experimental reporting rules and shared universal spectral reference databases, cross-laboratory data comparison and independent result validation remain difficult to implement.

Internal standardization is considered a viable approach to mitigate reproducibility defects. By introducing a fixed concentration of Raman-active internal reporters into samples, researchers can calibrate signal fluctuations originating from substrates and spectrometers [114,117]. Several studies have yielded relative standard deviations below 5% via this strategy, yet stable inter-laboratory performance has not been realized. Diverse internal standard candidates and inconsistent calibration workflows prevent the formation of a unified benchmark analogous to tetramethylsilane in NMR spectroscopy. Until reproducibility issues are fully addressed, SERS techniques will hardly gain official regulatory approval.

Complex food matrix interference acts as the second major constraint on practical detection. Natural pigments, lipids, and proteins in food samples generate intense fluorescence backgrounds and compete with target contaminants for hot-spot adsorption sites on plasmonic substrates, which undermines the accuracy and stability of quantitative SERS signals [134,150]. This interference is especially severe for mycotoxin detection in cereals and edible oils, where ultra-trace analyte concentrations are masked by massive matrix components [20]. The selectivity, non-specific adsorption, and cross-reactivity challenges inherent to SERS-based mycotoxin detection further complicate accurate quantification in these complex matrices [157]. Waxy layers and natural pigments on fruit and vegetable cuticles disrupt direct sampling and spectral acquisition for pesticide detection [46]. For heavy metal analysis in agricultural produce and drinking water, humic acids and inorganic salts trigger nonspecific metal-substrate adsorption and high background noise [16]. Although QuEChERS extraction and other purification protocols can weaken matrix interference [51], extra pretreatment steps sacrifice the core strength of SERS: rapid, on-site detection.

The third universal obstacle lies in the absence of unified testing standards and regulatory authorization. No global or regional food safety authority has released standardized operation specifications for SERS-based contaminant detection targeting pesticides, mycotoxins, or heavy metals. This gap is particularly evident across jurisdictions: the EU enforces strict default limits alongside comprehensive validation criteria (e.g., Decision 2002/657/EC), China implements full-fledged GB standards, and the US FDA maintains its dedicated method-validation guidance. As critically reviewed by Tsagkaris et al. [158], screening-oriented techniques such as SERS frequently report promising limits of detection acquired in simple buffer matrices, yet they commonly lack rigorous validation in real-world food commodities as well as benchmarking against established confirmatory methods—both are essential prerequisites for gaining regulatory acceptance. This calls for rigorous, reproducible end-to-end workflow validation aligned with well-established standards including ISO 17025 [159] and AOAC guidelines [160]. Great divergence in substrate fabrication, sample pretreatment, spectral acquisition and data analysis protocols blocks direct cross-laboratory result comparison and method validation. Apart from technical disparities, multiple non-technical barriers slow industrial adoption. Regulatory authorities usually require 5–10 years of systematic verification to authorize novel analytical techniques [134]. Food testing enterprises face high costs for new spectrometers, staff training and method validation if they abandon mature instruments such as LC-MS/MS, which have decades of validated data and established regulatory recognition [31]. Joint efforts from sensor developers, equipment manufacturers, regulators and end-users are required to break this industry inertia. Addressing these three cross-cutting bottlenecks, spectral irreproducibility, matrix interference, and the absence of standardized protocols, constitutes the central purpose of this review, and the solutions discussed above (inter-laboratory cross-testing, transfer learning, and XAI integration) represent the key pathways toward regulatory acceptance.

### 5.2. Prioritized Research Frontiers

To resolve the above cross-cutting limitations, targeted research should be carried out along several interconnected directions. Harmonized testing specifications and shared spectral databases are the foundational prerequisites for all subsequent technical improvements. The following research priorities correspond to the technical evolution rules summarized in Chapters 2–4, with supporting evidence including the supplementary sensing literature [28].

Unified experimental standards and open spectral reference libraries represent the most urgent research demand [17]. The international research community needs mutually agreed guidelines covering substrate manufacturing, spectral collection and data processing pipelines. Publicly accessible Raman reference datasets, similar to those for conventional vibrational spectroscopy, will help identify spectral artifacts and facilitate inter-group data alignment [98]. Multivariate optimization-based substrate design can effectively narrow performance gaps between different batches [43]. Developing certified SERS substrate reference materials and launching inter-laboratory joint testing programs will accelerate the formulation of universal validation criteria [110]. Flexible film and paper-based plasmon substrates recorded in supplementary papers provide low-cost solutions to produce evenly distributed hot spots for standardized field detection [25].

Portable field SERS platforms serve as a critical bridge between laboratory research and on-site screening [92]. Handheld Raman devices combined with optimized plasmon substrates and embedded chemometric algorithms enable contaminant detection without centralized laboratory facilities [85]. PDMS-cellulose composite flexible substrates allow direct swab sampling on irregular crop surfaces and simplify on-site pretreatment steps [25,43]. Future work should enhance equipment durability, extend battery life and optimize human–machine interfaces, alongside reducing substrate batch-to-batch signal variation under field conditions [92].

Interpretable machine learning algorithms are vital to raise detection accuracy and promote regulatory acceptance. Deep learning tools have achieved over 99 percent classification accuracy for mixed pesticide spectra via signal deconvolution [11,12]. A broader perspective on the integration of machine learning with nanomaterial-based optical sensors has highlighted the potential of algorithms such as support vector machines and random forests for enhancing the detection of benzimidazole fungicides in complex food matrices [161]. Recent applications of machine learning for pesticide analysis have further demonstrated that feature extraction and model validation strategies are critical for handling heterogeneous datasets and achieving reliable predictions in real-world samples [31]. Feature screening algorithms such as CARS and SPA eliminate redundant spectral variables and lower computing loads for miniature portable devices [52]. Nevertheless, most deep learning models operate as opaque black boxes, a fact that hinders regulatory evaluation and mechanistic interpretation of detection responses [11]. Explainable AI (XAI) toolkits such as SHAP and LIME provide systematic solutions to interpret black-box prediction outputs. Furthermore, rigorous model-validation protocols including stratified k-fold cross-validation and testing on fully external independent datasets should become standard practice to avoid over-optimistic performance estimates and improve model generalizability [34,35,36,37]. A feasible optimization route integrates DFT-simulated Raman spectra as physical constraints for neural network training. Hybrid models combining simulated and measured data deliver transparent prediction logic for mixed pesticide samples. Future algorithms should incorporate attention modules and saliency visualization to clarify which spectral peaks drive quantitative outputs, thus gaining wider recognition from regulatory institutions [12]. Chemometric tools optimized for complex matrix noise suppression, as documented in supplementary literature, can be embedded into portable sensing hardware [50]. Beyond analytical performance, the regulatory feasibility of AI-assisted food safety systems increasingly depends on legal and ethical governance. Recent analyses highlight that existing food safety regulations remain inadequately prepared for AI-assisted systems, creating gaps in interoperability, validation, liability, and algorithmic accountability. The same framework emphasizes that explainable algorithms and robust validation strategies are prerequisites for trustworthy AI-assisted risk assessment and decision-making [162]. Similarly, comprehensive reviews of AI in food testing conclude that trustworthy AI requires fairness, explainability, and systemic regulation, with XAI playing a central role in ensuring accountability [163]. Together, these perspectives reinforce that the integration of XAI into SERS-based workflows is not merely a technical enhancement but a necessary condition for regulatory acceptance and industrial translation.

Multi-analyte synchronous sensing chips cater to real-world food monitoring scenarios. Fresh agricultural and processed foods frequently contain coexisting pesticides, mycotoxins and heavy metals, yet most existing SERS sensors are designed for single-class contaminants [150]. Bimetallic core–shell nanoparticles coupled with multi-recognition probes enable simultaneous capture of diverse toxic substances in one measurement [29]. Microarray chips with independent recognition units separate overlapping Raman signals and realize multiplex quantification [70]. DNA- and aptamer-based signal amplification further lower detection limits for coexisting ultra-trace mycotoxins and heavy metals [103,119]. A competitive binding strategy has been developed for reliable signal-off SERS sensing of patulin in apples, achieving a detection limit of 0.02 ng/mL through programmable DNA interactions that regulate Raman signal intensity without affecting embedded Raman molecules [164]. The primary unresolved technical challenge is cross-reactivity between different recognition molecules and spectral overlap of multiple analytes [17]. Combined aptamer-antibody modification strategies can mitigate cross-interference and support parallel quantitative analysis [81]. Supplementary publications also provide multiplex sensing schemes for mixed fungicides on fruit surfaces [62,67].

Multi-spectral fusion techniques complement the respective strengths of SERS, NIR and hyperspectral imaging [18,30]. Near-infrared spectroscopy supports high-throughput, low-cost preliminary screening, hyperspectral imaging visualizes contaminant spatial distribution on food surfaces, and SERS provides ultra-trace confirmatory quantification [87,110]. Feature-level CNN fusion of NIR and SERS spectra improves aflatoxin quantitative precision in oil crops [18]. The potential of deep fusion strategies has been demonstrated by combining fluorescence hyperspectral imaging with NIR spectroscopy for the nondestructive detection of trace cadmium in lettuce leaves, where a CNN-based feature fusion method significantly outperformed single-spectrum models [165]. Similar fusion approaches have also been applied to predict selenium content in lettuce under cadmium-stressed environments, showcasing the versatility of multi-source data integration for heavy metal-related studies [166]. Joint analysis at feature or decision layers generally outperforms single-spectrum detection [18]. Practical implementation barriers include spatial alignment of different testing equipment and universal calibration models across instruments [137]. Dual-mode fluorescence-SERS sensors further strengthen anti-interference capacity for complicated food extracts [79]. With the popularization of miniature spectrometers and embedded computing chips, multi-spectral integrated systems will form tiered food safety monitoring workflows [134]. Photocatalytic plasmon substrates additionally realize simultaneous heavy metal identification and degradation after spectral testing, expanding the application scope of field sensors [118].

### 5.3. General Concluding Remarks

Over five decades, SERS has evolved from a fundamental optical phenomenon to a complete analytical detection system [7]. According to the distinct physicochemical properties of pesticides, mycotoxins and heavy metals, researchers have established differentiated sensing frameworks with tailored substrates and recognition elements [13]. Continuous substrate optimization, biological signal amplification and chemometric modeling have pushed detection limits from ppm down to fM levels [13,20]. Combined spectral preprocessing and modeling workflows support both qualitative identification and precise quantification of mixed pollutants in complex food matrices [31]. Supplementary research further explores flexible substrates, ligand-based probes and machine learning sensing strategies targeting all three contaminant categories [25,28,128].

A notable gap still separates ideal laboratory test outcomes from practical industrial testing requirements [134]. Spectral irreproducibility, severe matrix interference and absent unified testing standards are not minor technical defects but core bottlenecks restricting industrial translation [17,60]. Upcoming research should not merely pursue lower detection limits; it should also pay more attention to solving practical transformation obstacles [87]. Formulating unified testing specifications, developing interpretable artificial intelligence models and constructing multi-technique integrated monitoring platforms constitute three major directions to accelerate SERS industrialization [12].

A previously published review compared divergent SERS detection strategies for three dominant food contaminants, elaborating the intrinsic correlation between analyte physicochemical traits and sensing design, while summarizing three shared technical bottlenecks across all detection systems [13]. Mature substrate materials, biorecognition units and spectral processing algorithms can serve as viable solutions to these challenges [15]. The supplementary literature table collects a set of unintegrated advanced sensing approaches covering uniform hot-spot fabrication, anti-interference chemometrics and multiplex detection [11,25,30,43,67,70]. The central task for subsequent research is to convert discrete laboratory protocols into repeatable, standardized testing pipelines via cross-laboratory joint verification, so as to promote official regulatory acceptance of SERS for routine food safety surveillance [110].

## Figures and Tables

**Table 1:** Representative SERS-based methods for pesticide residue detection in food matrices.

Target Analyte	Food Matrix	SERS Substrate	Raman Peaks (cm^−1^)	Matrix Type	Reported LOD	Original Unit	Ref.
Ametryn	Apple, potato peels	rGO/AgNP self-assembled film	992	Spiked food matrix	22.77	μg/kg	[46]
Thiabendazole	Cherry peel	Au/Ag/G/PDMS flexible film	1002	Spiked food matrix	10	μg/kg	[47]
Chlorpyrifos, Flumetralin	Tobacco leaves	SiO_2_@Ag-SERS substrate	Carbendazim: 1227, 1274, 1461, 1517; Flumetralin: 1352	Spiked food matrix	0.1/1	mg/kg	[48]
Chlorpyrifos	Tomato	Silver colloid (~63 nm)	341, 412, 678, 1092, 1571	Spiked food matrix	0.8	μg/kg	[40]
Thiram + Paraquat	Green tea infusion	Au nanorod cluster fiber probe	Thiram: 1369; Paraquat: 1647	Spiked food matrix	1.0/10.0	μg/kg	[53]
Chlorpyrifos + Pyrimethanil	Apple, strawberry peels	Citrate-AuNPs (16.03 ± 0.93 nm)	Chlorpyrifos: 609, 675, 1092; Pyrimethanil: 558, 990, 1294, 1600	Spiked food matrix	2.1 × 10^−4^/1.2 × 10^−4^	μg/kg	[61]
Phosmet, Thiabendazole, Acetamiprid	Leafy vegetables	Seed-mediated AuNPs (~50 nm)	Phosmet: 500, 604, 710, 1012; TBZ: 784, 1008; Acetamiprid: 550, 634, 832	Spiked food matrix	<500–1000	μg/kg	[55]
Acephate, Carbendazim, Thiamethoxam, Tricyclazole	Basmati rice	Citrate-AuNPs (25.4–81.8 nm)	Acephate: 682; Carbendazim: 633, 1006; Thiamethoxam: 638; Tricyclazole: 1371	Spiked food matrix	800/0.6/0.7/0.8	μg/kg	[43]
TMTD, PYM, AC, TBZ, PQ, CBZ, ET, FEN, CTL)	Tomato juice	AgNS/AgNIF hybrid platform	TMTD: 560, 932, 1144, 1385, 1508; TBZ: 784; PQ: 1647; CBZ: 633, 1006	Standard solution	0.00387–6.26 (standard); 4.6 (TMTD in tomato)	M	[67]
10 pesticides (incl. TBZ)	Apple peel	Ag@BNNPs (one-pot synthesis)	TBZ: 784	Spiked food matrix	TBZ: 0.010	μg/kg	[57]

TBZ, thiabendazole; LOD, limit of detection; ppb, parts per billion; pM, picomolar; M, molar. “Not reported” indicates that the LOD value was not provided in the original reference. LOD values with the unit mg/cm^2^ represent surface density (mass per unit area) rather than mass concentration. Multiple target analytes in a single row indicate simultaneous detection. Spiked food matrix denotes samples spiked with target analytes; standard solution means measurements performed purely in buffer or solution without real-food matrix.

**Table 2:** Representative SERS-based methods for mycotoxin detection in food matrices.

Target Analyte	Food Matrix	SERS Substrate	Recognition & Amplification Strategy	Matrix Type	Reported LOD	Original Unit	Ref.
AFB1	Peanut oil, rice	AuNPs/Si wafer (70 ± 5 nm)	Immunoassay; oriented antibody immobilization	Spiked food matrix	0.2	pg/mL	[74]
AFB1	Rice, corn, wheat	Fe_3_O_4_@Au + Au nanostars	Aptamer; core-satellite self-assembly	Spiked food matrix	0.24	pg/mL	[76]
AFB1	Peanut, corn, almond	AgNPs (hydroxylamine-reduced)	CRISPR/Cas12a + G4-DNAzyme; TMB-TMBox substrate	Spiked food matrix	0.79	pg/mL	[3]
AFB1	Milk, soy sauce	Au@PB@Au core–shell NPs	CRISPR-Cas12a + EXO I	Spiked food matrix	3.55	pg/mL	[84]
DON	Corn, wheat	Citrate-AuNPs (24 ± 6 nm)	Label-free; DFT-assisted peak assignment	Standard solution	0.0085	μg/mL	[91]
DON	Corn, wheat	Rh@Ag@PBNPs	Dual-mode LFIA (colorimetric/SERS); KNN/ANN prediction	Spiked food matrix	4.21	pg/mL	[92]
ZEN	Corn, soybean, rice flours	BNC/SNP + Au nanostars	Aptamer; 3D flexible core-satellite “turn-off” assembly	Spiked food matrix	1.40	pg/mL	[93]
AOH	Kiwi, tomato, citrus	Au nanoflowers	FL-SERS dual-channel aptasensor; opposite signal responses	Spiked food matrix	0.064	ng/mL	[94]
FB1	Corn, wheat, onion, skim milk	Ag-pSi	Ratiometric aptasensor; 4-ATP IS; 5-regenerable substrate	Spiked food matrix	0.05	ppb	[95]
AFB1, FB1, OTA	Corn, rice, wheat	Ag-pSi microarray	Aptamer-based multiplex; DTNB reporter; 25-spot high-throughput; regenerable	Spiked food matrix	AFB1: 0.008; FB1: 0.547; OTA: 0.922	ppb	[70]

AFB1, aflatoxin B1; DON, deoxynivalenol; ZEN, zearalenone; AOH, alternariol; FB1, fumonisin B1; OTA, ochratoxin A; PATP, p-aminothiophenol; 4-MBA, 4-mercaptobenzoic acid; 4-ATP, 4-aminothiophenol; IS, internal standard; ppb, parts per billion; pM, picomolar. Multiple target analytes in a single row indicate simultaneous detection. Spiked food matrix denotes samples spiked with target analytes.

**Table 4:** SERS LODs and regulatory limits across contaminant classes.

Contaminant Class	Analyte (Matrix)	MPC (ppb)	SERS LOD (ppb)	LOD (Buffer)	Safety Margin	Ref.
Pesticides	Chlorpyrifos (Wheat)	500 ^a^	0.558	n.r.	~896-fold	[56]
	Thiram (Tea soup)	≤10 (Banned) ^a,b^	1.0	n.r.	10-fold	[53]
	Paraquat (Tea soup)	200 ^a^	10.0	n.r.	20-fold	[53]
	Carbendazim (Basmati rice)	10 ^c^	<10 ^d^	n.r.	>1-fold	[59]
	Tricyclazole (Basmati rice)	10 ^c^	<10 ^d^	n.r.	>1-fold	[47]
Mycotoxins	AFB1 (Peanut, corn, almond)	20 ^e^	0.00079	n.r.	~25,000-fold	[3]
	DON (Corn, wheat)	1000 ^e^	8.5	n.r.	~117-fold	[91]
	ZEN (Corn, soybean, rice flours)	60 ^e^	0.0014	n.r.	~42,000-fold	[93]
	FB1 (Corn, wheat, onion, skim milk)	1000 (EU) ^f^	0.05	n.r.	~20,000-fold	[95]
	AFB1 (Corn, rice, wheat)	20 ^e^	0.008	n.r.	~2500-fold	[70]
Heavy Metals	Pb^2+^ (Tea powder, glutinous rice flour)	500 ^g^	0.021	n.r.	~2.4 × 10^4^-fold	[109]
	Hg^2+^ (Fish meat samples)	500 ^h^	~0.027 ^i^	n.r.	~1.9 × 10^4^-fold	[112]
	Cd^2+^ (Black tea powder)	300 ^g^	0.0000236	n.r.	~1.3 × 10^7^-fold	[115]
	Total Arsenic (Keemun black tea)	2000 ^g^	5.61	n.r.	~356-fold	[116]
	As^3+^/As^5+^ (Rice)	200 ^j^	~0.0013/~0.000067 ^i^	n.r.	~1.5 × 10^5^-fold	[126]

LOD, limit of detection; MPC, maximum permissible concentration. ^a^ GB 2763-2026 [129]. ^b^ Banned in Chinese tea production; EU default limit used. ^c^ EU regulations. ^d^ QuEChERS-induced aggregation lowered LOD below 10 ppb. ^e^ GB 2761-2017 [130]. ^f^ No national limit; EU reference used. ^g^ GB 2762-2022 [131]. ^h^ GB 2762-2022 (methylmercury). ^i^ Molar concentrations converted to ppb. ^j^ GB 2762-2022 (inorganic arsenic). LOD (buffer) indicates the LOD determined in pure buffer or standard solution, when reported; n.r. = not reported. Safety margin = MPC/LOD.

## Data Availability

No new data were created or analyzed in this study. Data sharing is not applicable to this article.

## Abbreviations

The following abbreviations are used in this manuscript:

4-ATP	4-Aminothiophenol
4-MBA	4-Mercaptobenzoic acid
4-MPY	4-Mercaptopyridine
AAS	Atomic Absorption Spectrometry
AFB1	Aflatoxin B1
AFS	Atomic Fluorescence Spectrometry
ANN	Artificial Neural Network
CARS	Competitive Adaptive Reweighted Sampling
CNN	Convolutional Neural Network
CRISPR	Clustered Regularly Interspaced Short Palindromic Repeats
DDA	Discrete Dipole Approximation
DFT	Density Functional Theory
DON	Deoxynivaleno
ELISA	Enzyme-Linked Immunosorbent Assay
EM	Electromagnetic enhancement
FB1	Fumonisin B1
FCNN	Fully Connected Neural Network
FDTD	Finite-Difference Time-Domain
GC	Gas Chromatography
HPLC	High-Performance Liquid Chromatography
HSI	Hyperspectral Imaging
ICP-MS	Inductively Coupled Plasma Mass Spectrometry
ICP-OES	Inductively Coupled Plasma Optical Emission Spectrometry
IS	Internal Standard
LC	Liquid Chromatography
LDA	Linear Discriminant Analysis
LOD	Limit of Detection
LSPR	Localized Surface Plasmon Resonance
MIPs	Molecularly Imprinted Polymers
MS	Mass Spectrometry
NIR	Near-Infrared Spectroscopy
OTA	Ochratoxin A
PCA	Principal Component Analysis
PCR	Principal Component Regression
PLS	Partial Least Squares
PLS-DA	Partial Least Squares Discriminant Analysis
PLSR	Partial Least Squares Regression
QuEChERS	Quick, Easy, Cheap, Effective, Rugged, and Safe
R6G	Rhodamine 6G
RhB	Rhodamine B
SERS	Surface-Enhanced Raman Spectroscopy
SIMCA	Soft Independent Modeling of Class Analogy
SPA	Successive Projection Algorithm
TMT	Trithiocyanuric acid
ZEN	Zearalenone
